# Gene expression profiling of Jack Pine (*Pinus banksiana*) under copper stress: Identification of genes associated with copper resistance

**DOI:** 10.1371/journal.pone.0296027

**Published:** 2024-03-07

**Authors:** Alistar Moy, Karolina Czajka, Paul Michael, Kabwe Nkongolo

**Affiliations:** 1 Biomolecular Sciences Program, School of Natural Sciences, Laurentian University, Sudbury, Ontario, Canada; 2 Department of Biology, School of Natural Sciences, Laurentian University, Sudbury, Ontario, Canada; National Botanical Research Institute CSIR, INDIA

## Abstract

Understanding the genetic response of plants to copper stress is a necessary step to improving the utility of plants for environmental remediation and restoration. The objectives of this study were to: 1) characterize the transcriptome of Jack Pine (*Pinus banksiana*) under copper stress, 2) analyze the gene expression profile shifts of genotypes exposed to copper ion toxicity, and 3) identify genes associated with copper resistance. *Pinus banksiana* seedlings were treated with 10 mmoles of copper and screened in a growth chamber. There were 6,213 upregulated and 29,038 downregulated genes expressed in the copper resistant genotypes compared to the susceptible genotypes at a high stringency based on the false discovery rate (FDR). Overall, 25,552 transcripts were assigned gene ontology. Among the top upregulated genes, the response to stress, the biosynthetic process, and the response to chemical stimuli terms represented the highest proportion of gene expression for the biological processes. For the molecular function category, the majority of expressed genes were associated with nucleotide binding followed by transporter activity, and kinase activity. The majority of upregulated genes were located in the plasma membrane while half of the total downregulated genes were associated with the extracellular region. Two candidate genes associated with copper resistance were identified including genes encoding for heavy metal-associated isoprenylated plant proteins (AtHIP20 and AtHIP26) and a gene encoding the pleiotropic drug resistance protein 1 (NtPDR1). This study represents the first report of transcriptomic responses of a conifer species to copper ions.

## Introduction

Understanding plant resistance to copper is an important step to efficiently revitalize areas afflicted by mining and industrial pollution. Plant resistance to copper is especially important for areas that are poised to increase copper production and exportation. Many facets of plant development and physiology are reliant on copper and the role it plays as an essential ion. Proteins that utilize copper are associated with photosynthesis, cellular respiration, cell wall fortification, growth modulation, apoptosis, and antioxidative functions [1–4]. An excess of copper causes a variety of symptoms that may damage tissues and impede development. Excess of copper in root cells, competitively inhibits the uptake of essential ions such as iron, manganese, and zinc resulting in disturbed ion homeostasis [5–7]. It can replace iron in the binding site of plastoquinone QA of Photosystem II, leading to diminished electron transfer during photosynthesis [8]. Copper toxicity leads to decreased chlorophyll and thylakoid membranes content, which impedes photosynthesis and contribute to chlorosis [9]. Decreased nitric oxide production is another symptom of copper toxicity that results in diminished auxin production, cytokinin activity, and mitotic activity in root cells. ROS production induced by copper may also lead to oxidative stress, lipid peroxidation, plant tissue damage and organelle death [10–13].

The genetic and physiological bases of copper resistance have been partially described in the literature. In *Arabidopsis thaliana*, the downregulation of COPT1, COPT2, ZIP2 and ZIP4 indicate a decrease in the initial uptake of copper into the root cells [14–16]. Upregulation of the HMA5 transporter in *Arabidopsis thaliana* and *Oryza sativa* suggests an increased copper mobilization in roots and also an increased root to shoot Cu translocation [15, 17, 18]. An upregulation of HMA1 and HMA6/PAA1 transporters in *Arabidopsis thaliana*, suggests an increased of root to shoot Cu translocation and its transport to chloroplasts [15, 19, 20]. The HMA8/PAA2 transporter was also upregulated, demonstrating the increased transport of copper to the thylakoid lumen and plastocyanin [15, 21, 22]. Collectively, the higher amount of Cu delivery to the chloroplast stroma, thylakoid lumen and plastocyanin may suggest increased photosynthesis [22, 23]. Similarly, the increased expression of AtHMA7 encourages copper transport to the Golgi apparatus and ethylene receptors located in the ER, suggesting an enhanced growth to counteract Cu toxicity symptoms [24]. The OsHMA9 gene was upregulated in the xylem and phloem of *Oryza sativa* exposed to Cu indicating an increased xylem and phloem loading [20]. Genes encoding the chelators MT2a and MT2b were upregulated in root tips, shoots, and phloem area which indicated an elevated metallothionein production for copper chelation in those respective areas [25, 26].

Currently, the response of conifers to metals are elusive and under researched in comparison to angiosperms. Transcriptome analysis of copper resistant trees will be a valuable tool to uncover physiological mechanisms associated with copper resistance or tolerance. *Pinus banksiana* was selected as a candidate for transcriptome analysis due to its successful utilization in the Sudbury regreening program [27–29]. In addition to being a hardy and resilient tree, *Pinus banksiana* was observed to have a moderate genetic diversity and low gene flow in metal contaminated sites [30, 31]. Furthermore, newer population of *Pinus banksiana* that were used in the regreening program had a significantly higher genetic diversity in comparison to older populations.

The objectives of this study were to: 1) characterize the transcriptome of Jack Pine (*Pinus banksiana*) under copper stress, 2) analyze the gene expression variation in genotypes exposed to copper ion toxicity and 3) identify genes associated with copper resistance.

## Materials and methods

### Plant treatment

*Pinus banksiana* seedings were provided by the College Boreal Plant Center located in Sudbury, Ontario. Six-month old seedlings of *Pinus banksiana* were grown in growth chambers at Laurentian University according to the methodology outlined in Moarefi and Nkongolo (2022) [32]. Initially, they were transplanted into planter pots containing a 1:1 mixture of sand and a mixture of 79% Sphagmum moss, 17% perlite, 5% composted peatmoss. The seedlings were then incubated in a growth chamber for one month. They were fertilized with a 1:1:1 mixture of nitrogen, phosphorous, and potassium fertilizer when required. After this one month period, fifteen seedlings were treated with 1,300 mg of copper sulphate per 1 kg of soil (10 mmol of Cu). This treatment represented the in field concentration of Cu from a survey on metal contaminated sites in the Greater Sudbury Region [29]. Ten seedlings were treated with deionized water that represented the negative control. Ten seedlings were treated with a molar equivalent of potassium sulphate relative to copper sulphate with respect to the sulphate ions. Damages were measured 14 days after treatment using a 1 to 9 scale, 1 indicating no damage and 9 indicating dead plants as described in Moarefi and Nkongolo (2022) [32]. Details of the rating scale is described in S1 Table in S1 File. Needles from resistant genotypes with no symptoms and green needles from susceptible genotypes were harvested and wrapped in individual aluminum foils for RNA extraction. For longer term storage, the needles were flash frozen using liquid nitrogen and stored in a freezer at -80°C.

### RNA extraction

Transcriptome analysis was performed on three plants (3 replicates) for each treatment (copper sulfate, potassium sulfate, and water) and for each type of the genotypes (resistant and susceptible). RNA extraction was performed on the green needles of the seedlings following the protocol from the NORGEN BIOTEK Plant/fungi total RNA purification Kit https://norgenbiotek.com/product/plantfungi-total-rna-purification-kit. Agarose gel electrophoresis was performed on the extracted RNA to assess RNA quality. The quantity of RNA for each sample was determined using the Qubit™ RNA BR assay kit. The extracted RNA samples were stored in a freezer at -80°C.

### RNA sequencing and de novo transcriptome assembly

Messenger RNA (mRNA) was isolated from total RNA. RNA chemical fragmentation was done to account for the size limitation of the sequencing platform. Then, this mRNA was reverse transcribed to cDNA using reverse transcriptase and RNAse was added to prevent unnecessary ligation between different nucleotide strands. Second strand synthesis was performed followed by 3’ end ligation with adaptors and adenosine caps. The cDNA was amplified to generate cDNA libraries. Illumina sequencing (performed at Seqmatic in San Francisco, California, USA) was used to sequence the cDNA libraries. FASTQC files containing the raw reads of each cDNA library was generated for each sample. The FASTQC program verified the quality of raw data from the files and provided attributes for each sequence which included average sequence length, % GC content, total deduplicated percentage and sequences flagged as poor quality. The Cutadapt program was used to remove adaptor sequences and low-quality bases from the raw read data. The Bowtie2 algorithm in Trinity was used to map RNA sequence raw reads to the trinity transcript assembly, generating sequence alignment map (SAM) files which were then converted to BAM (binary form of SAM) files. Transcript assembly was performed by inputting RNA sequence data from all samples into the TRINITY program, which quantified the number of genes based on the number of detected isoforms.

### BLAT matching and annotation of *Pinus banksiana* genes

Transcripts were characterized by performing a two-way BLAST-like alignment tool (BLAT) matching with the *Pinus taeda* genome as a reference. Attributes such as Transcript ID, Gene ID, and corresponding log (E-value) for sequence similarity with the reference genome was characterized. Other characteristics identified by BLAT matching include query sequence size, transcript sequence size, and the percentage of net match for each characteristic. Transcripts were mapped to protein sequences in the UniProt database, generating corresponding UniProt IDs. Protein matches with the highest degree of similarity were used to annotate genes and assign gene ontology information such as gene description.

### Quantification of gene expression and quality control (QC) analysis

The RNA-Seq by Expectation-Maximization (RSEM) abundance estimation method was used to quantify the expression level of each gene/transcript and related isoforms. Quality control for read count was performed to critically assess the number of counts from each gene. Raw reads were filtered and selected for counts of at least 1, 2, 10, 50 or 100. Genes with one read were considered noise. Genes with two or more counts were used as an estimate for the number of genes expressed. Genes with 10 or more counts were considered an adequate indication of the number of genes that had enough reads for downstream statistical analysis. For each treatment group, genes with a counts per million (CPM) value of 1 or higher in at least 2 samples were included in downstream analysis. Genes with a CPM value of less than 1 in at least 2 samples were unexpressed and removed. Normalization factors for raw counts were generated using a trimmed mean of M-values (TMM) from edge R to remove variations from samples and normalize the samples.

The normalized read counts were log-scale transformed using the voom method (log2 scale) from the R limma package. Boxplots of the transformed expression values were generated to show the mean distribution of every sample. Deviation from the mean distribution in a particular sample may indicate variations among experimental conditions, sample contamination or batch effect. Samples that deviated significantly from the mean distribution within the same objective group were excluded.

Multidimensional scaling plots were generated to display the clustering of sample groups based on the leading logFC of normalized data. Groups of samples that deviated significantly from other groups of samples were considered differentially regulated. Samples that deviated significantly from the other samples within the same group were considered outliers and not included in downstream analysis.

A heatmap was generated from the logFC of 5000 genes to show the relationship of gene expression between the samples. Samples that did not have a similar logFC were considered outliers and were not included in downstream analysis. The proportion of raw reads expressed by the top 100 upregulated and downregulated genes were also assessed in every sample to identify potential bottlenecking issues.

### Differential gene expression (DGE) analysis of pairwise comparisons

The cut-off for pairwise comparisons was calculated to be equivalent to 10 raw counts. From the average of total counts in all samples, a CPM of 0.361 was calculated as the minimum threshold required in pairwise comparisons. Genes that had a CPM higher than the cutoff in at least two samples were included in downstream analysis whereas genes that did not fulfill these parameters were excluded. The pairwise comparisons of transcripts were performed between treated samples and the control. Differential gene expression expressed as logFC values were evaluated using the R limma package. To assess the interference of sulphate ions on the treatment regimen, pairwise comparisons of expressed genes were also conducted between copper treated plants and the potassium and water used as controls. The entire set of genes for each pairwise comparison was annotated using Trinotate and Trinity. Gene ontology was performed by assigning GO terms and gene IDs from available databases to the set of genes for a particular pairwise comparison. Genes that could not be annotated were filtered out of the set of annotated genes. Each gene set was run through a plant slim function using the Omicsbox program. Gene ontology charts functionally categorizing biological process, metabolic function, and cellular component were generated. For each functional category, sequences were distributed using the NodeScore of each assigned GO term.

### Analysis of top differentially regulated genes

The top 100 upregulated and downregulated genes were ranked between copper treated plants and the control. Genes were ranked based on the LogFC and the fulfillment of high stringency parameters. UniProt annotation and review of the current literature was done to characterize genes associated with copper detoxification tolerance mechanisms. Genes associated with copper resistance were considered candidate genes. Gene ontology charts functionally categorizing biological processes, metabolic functions, and cellular component localization were generated for the top 100 regulated genes using the aforementioned process in DGE analysis. Charts comprised of the top 25 genes were provided for each pairwise comparison.

The top 100 genes for each pairwise comparison was obtained from the set of differentially expressed genes and categorized as upregulated or downregulated. Genes with the highest or lowest expression were correlated to copper stress and can be used to partially describe the genetic response to copper. Protein descriptions with the “predicted protein” label indicated no assignment of any closely related protein or relevant GO terms from the UniProt database. Gene ontology terms and functional categorizations were assigned by the Omics Box/BLAST2GO program.

## Results

### Transcript assembly and QC analysis of sequences

The FastQC program was used to analyze the raw reads generated from Illumina sequencing. No sequences were flagged as poor quality. Copper resistant plants had 31–49 million sequences and copper susceptible plants had 21–29 million sequences. Both treatment groups had an average sequence length of 51 bases. The trinity program facilitated transcript assembly, producing 581,037 transcripts. Overall, 78–83% of genes were mapped to the database for copper resistant individuals, and 62% for copper susceptible genotypes. A total of 126,460 genes fulfilled the CPM related parameters and were used for differential gene expression analysis.

### Differential gene expression (DGE) analysis between genotypes

**Fig 1:**
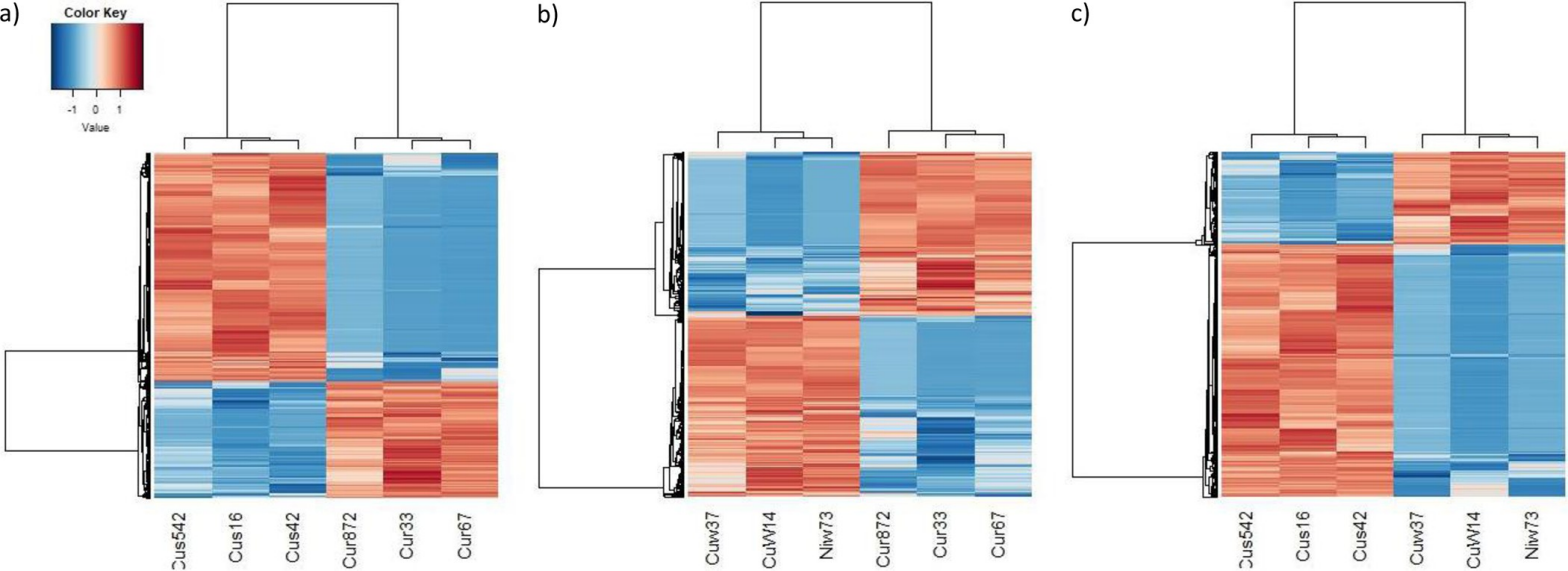
Heatmap of differentially expressed genes in *Pinus banksiana* a) Copper resistant genotype compared to copper susceptible genotype; b) Copper resistant genotype compared to water control; and c) Copper susceptible genotype compared to water control. Differentially expressed gene values are based on the Log2 normalized FC, with red cells representing upregulation and blue cells representing downregulation. Cus542, Cus16 and Cus42 are copper susceptible samples. Cur872, Cur33 and Cur67 are copper resistant samples. Cuw37, CuW14 and Niw73 are the water controls.

**Fig 2:**
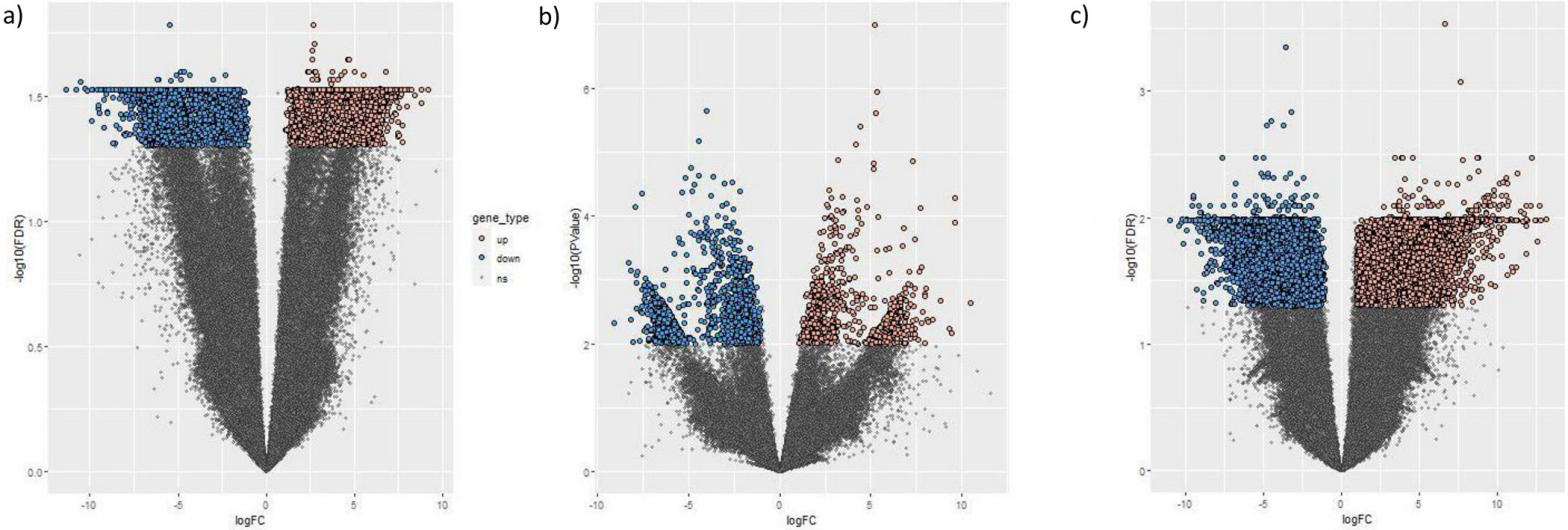
Volcano plot of differentially expressed genes from *Pinus banksiana*: a) Copper resistant genotype compared to copper susceptible genotype; b) Copper resistant genotype compared to water controls; and c) Copper susceptible genotype compared to water controls. Brown points represent upregulated gene expression whereas blue points represent downregulated gene expression when compared to the susceptible genotype. Grey points indicate no significant difference from the susceptible genotypes. Log10(FDR) is the log10 of the false discovery rate. The border between the no significant points and the differentially regulated genes represents the false discovery rate of 0.05 (two fold).

This transcriptome shotgun assembly project has been deposited in the NCBI BioProject database with the accession number PRJNA962116. The clustering among samples was visually assessed using a multidimensional scale plot and hierarchical cluster map. The water and potassium control groups clustered within the same region, indicating no significant difference in gene expression between the treatment groups. The presence of the potassium control did not significantly affect the treatment regimen. Clustering between the resistant genotype (RG) and susceptible genotype (SG) was low, indicating that gene expression was significantly different between the two groups. In fact, 6,213 genes were upregulated and 29,038 genes were downregulated when RG were compared to SG (Table 1). For RG and the water control, clustering occurred within the same region and overlapped, demonstrating a similar pattern of gene expression. Only one gene was upregulated when RG wee compared to water control. No clustering was observed between SG and the water control. DEGs that fulfilled the high stringency cut off (two fold and FDR 0.05) were considered for downstream DGE analysis. The heatmaps and the volcano plots of the differentially expressed genes for copper RG compared to copper SG, RG compared to water, and SG compared to water are illustrated in Figs 1 and 2.

**Table 1:** Differentially expressed genes from the copper resistant genotype compared to the copper susceptible and water and copper susceptible genotypes compared to water in *Pinus banksiana*.

Genotype	Gene expression	*FDR	P-value
Resistant vs susceptible	Upregulated	6213	6431
	Downregulated	29038	29605
Resistant vs water	Upregulated	1	1138
	Downregulated	0	1250
Susceptible vs water	Upregulated genes	27584	31972
	Downregulated genes	10065	12130

*False discovery rate (FDR)

### Gene ontology of the top 100 differentially expressed genes for *Pinus banksiana*

Overall, 581,037 transcripts were mapped to protein sequences in the UniProt database and the closest matches were used to annotate genes. Overall, 25,552 transcripts were assigned gene ontology. Tables 2 to 3 show the top 25 upregulated and downregulated genes when copper RG were compared to SG and Tables 4 and 5 when SG were compared to water. Extended data including the top 50 upregulated and downregulated genes when RG and SG, and SG and controls were compared and are described in Tables 2S to 5S in S1 File. More importantly, Table 6 described the two candidate genes associated with copper resistance in *P*. *banksiana*.

**Table 2:** Top 25 upregulated genes from copper resistant samples compared to copper susceptible samples in *Pinus banksiana*.

Rank	Gene ID	Res 1	Res 2	Res 3	Sus 1	Sus 2	Sus 3	logFC	Adj. P. Value	UniProt Description
**0**	TRINITY_DN35689_c0_g1	12.05	7.41	11.07	0	0	0	9.21	0.00002	Predicted Protein
**1**	TRINITY_DN10618_c0_g1	17.42	2.72	9.6	0	0	0	8.82	0.00181	Predicted Protein
**2**	TRINITY_DN91621_c0_g2	13.93	4.82	5.68	0	0	0	8.78	0.00025	Predicted Protein
**3**	TRINITY_DN199894_c0_g2	9.44	9.02	1.99	0	0	0	8.45	0.00117	Predicted Protein
**4**	TRINITY_DN28042_c0_g3	4.91	8.95	2.74	0	0	0	8.26	0.00022	Cytochrome P450 750A1, EC 1.14.-.- (Cytochrome P450 CYPC)
**5**	TRINITY_DN7900_c0_g1	6	6.27	3.22	0	0	0	8.25	0.00007	Predicted Protein
**6**	TRINITY_DN2617_c0_g1	8.29	4.5	3.27	0	0	0	8.24	0.00017	Predicted Protein
**7**	TRINITY_DN20922_c0_g1	3.17	6.13	4.05	0	0	0	8.02	0.00004	Predicted Protein
**8**	TRINITY_DN236262_c0_g1	11.08	1.85	3.41	0	0	0	7.96	0.00197	Predicted Protein
**9**	TRINITY_DN95006_c0_g1	6.22	4.28	1.99	0	0	0	7.87	0.00033	Predicted Protein
**10**	TRINITY_DN219929_c1_g1	2.74	4.62	4.69	0	0	0	7.86	0.00003	Predicted Protein
**11**	TRINITY_DN4529_c0_g1	3	7.73	1.66	0	0	0	7.73	0.00069	1,8-cineole synthase, chloroplastic, EC 4.2.3.108 (Terpene synthase TPS-Cin, PgTPS-Cin)
**12**	TRINITY_DN13781_c0_g3	16.16	15.27	4.31	0	0	1.35	7.73	0.00318	Predicted Protein
**13**	TRINITY_DN216_c0_g1	58.07	22.76	12.46	0.07	0.49	0.97	7.72	0.00440	Fatty acyl-CoA reductase 2, chloroplastic, AtFAR2, EC 1.2.1.84 (Fatty acid reductase 2) (Male sterility protein 2)
**14**	TRINITY_DN9994_c0_g1	8.13	1.94	2.38	0	0	0	7.67	0.00114	Predicted Protein
**15**	TRINITY_DN5226_c2_g1	3.74	2.06	5.32	0	0	0	7.66	0.00014	Predicted Protein
**16**	TRINITY_DN57458_c1_g1	2.43	3.07	4.49	0	0	0	7.58	0.00004	Predicted Protein
**17**	TRINITY_DN3869_c0_g1	3.23	4.31	2.12	0	0	0	7.58	0.00009	Predicted Protein
**18**	TRINITY_DN47098_c0_g1	7.77	2.08	1.81	0	0	0	7.58	0.00131	Predicted Protein
**19**	TRINITY_DN19214_c1_g2	3.44	3.21	2.82	0	0	0	7.57	0.00004	Predicted Protein
**20**	TRINITY_DN40558_c0_g2	1.22	7.95	3.16	0	0	0	7.57	0.00127	Predicted Protein
**21**	TRINITY_DN18490_c0_g1	2.06	7.43	1.88	0	0	0	7.57	0.00066	Predicted Protein
**22**	TRINITY_DN9649_c1_g3	0.84	8.97	3.91	0	0	0	7.54	0.00382	Predicted Protein
**23**	TRINITY_DN148688_c0_g2	6.51	3.72	1.03	0	0	0	7.54	0.00203	Predicted Protein
**24**	TRINITY_DN100_c0_g2	7.29	15.7	4.96	0	0.41	0.08	7.53	0.00072	Predicted Protein
**25**	TRINITY_DN178176_c0_g1	3.36	4.4	1.77	0	0	0	7.53	0.00017	Predicted Protein

**Table 3:** Top 25 downregulated genes from copper resistant samples compared to copper susceptible samples in *Pinus banksiana*.

Rank	Gene ID	Res 1	Res 2	Res 3	Sus 1	Sus 2	Sus 3	logFC	Adj. P. Value	UniProt Description
**0**	TRINITY_DN3519_c0_g1	0	0	0	191.35	54.37	115.22	-11.34	0.00021	Predicted Protein
**1**	TRINITY_DN43547_c0_g1	0	0	0	162.58	38.33	61.13	-10.81	0.00035	Predicted Protein
**2**	TRINITY_DN2824_c0_g1	0	0.03	0	90.93	154.91	84.36	-10.53	0.00000	Polygalacturonase, PG, EC 3.2.1.15 (Pectinase)
**3**	TRINITY_DN2824_c0_g1	0	0.03	0	90.93	154.91	84.36	-10.53	0.00000	Probable polygalacturonase At1g80170, PG, EC 3.2.1.15 (Pectinase At1g80170)
**4**	TRINITY_DN1315_c0_g1	0.43	0.06	0.31	763.2	1448.5	490.5	-10.28	0.00001	Beta-glucosidase 12, EC 3.2.1.21
**5**	TRINITY_DN1315_c0_g1	0.43	0.06	0.31	763.2	1448.5	490.5	-10.28	0.00001	Furcatin hydrolase, FH, EC 3.2.1.161
**6**	TRINITY_DN1315_c0_g1	0.43	0.06	0.31	763.2	1448.5	490.5	-10.28	0.00001	Non-cyanogenic beta-glucosidase, EC 3.2.1.21
**7**	TRINITY_DN1315_c0_g1	0.43	0.06	0.31	763.2	1448.5	490.5	-10.28	0.00001	Beta-glucosidase 27, Os8bglu27, EC 3.2.1.21
**8**	TRINITY_DN1315_c0_g1	0.43	0.06	0.31	763.2	1448.5	490.5	-10.28	0.00001	Beta-glucosidase 11, Os4bglu11, EC 3.2.1.21
**9**	TRINITY_DN1315_c0_g1	0.43	0.06	0.31	763.2	1448.5	490.5	-10.28	0.00001	Beta-glucosidase 24, Os6bglu24, EC 3.2.1.21
**10**	TRINITY_DN1315_c0_g1	0.43	0.06	0.31	763.2	1448.5	490.5	-10.28	0.00001	Beta-glucosidase 13, Os4bglu13, EC 3.2.1.21
**11**	TRINITY_DN67935_c0_g1	0	0	0	16.39	97.3	73.99	-10.13	0.00080	Predicted Protein
**12**	TRINITY_DN702_c0_g1	0	0.03	0	59.4	116.45	27.95	-9.93	0.00006	Cytochrome P450 71AU50, EC 1.14.-.-
**13**	TRINITY_DN702_c0_g1	0	0.03	0	59.4	116.45	27.95	-9.93	0.00006	Cytochrome P450 750A1, EC 1.14.-.- (Cytochrome P450 CYPC)
**14**	TRINITY_DN2358_c0_g1	0	0	0	81.45	89.01	7.38	-9.91	0.00287	Predicted Protein
**15**	TRINITY_DN31159_c0_g1	0	0	0	32.73	41.22	41.42	-9.83	0.00001	Predicted Protein
**16**	TRINITY_DN157611_c0_g2	0	0	0	22.19	59.12	26.75	-9.60	0.00002	Predicted Protein
**17**	TRINITY_DN10725_c0_g1	0	0	0	29.76	70.11	14.01	-9.55	0.00010	Predicted Protein
**18**	TRINITY_DN30360_c0_g2	0	0	0	98.39	13.66	18.36	-9.53	0.00190	Predicted Protein
**19**	TRINITY_DN251401_c0_g1	0	0	0	93.47	11.8	22.49	-9.52	0.00209	Predicted Protein
**20**	TRINITY_DN27632_c0_g2	0	0	0	8.22	77.05	47.39	-9.45	0.00238	Predicted Protein
**21**	TRINITY_DN10160_c0_g1	0	0	0	14.16	73.48	23.65	-9.41	0.00024	Predicted Protein
**22**	TRINITY_DN1453_c1_g4	0	0	0	36.74	50.95	10.28	-9.37	0.00016	Predicted Protein
**23**	TRINITY_DN57079_c0_g1	6.63	0	0	223.25	327.76	184.27	-9.36	0.00049	Predicted Protein
**24**	TRINITY_DN3979_c0_g1	0.05	0.01	0	52.76	91.37	16.98	-9.28	0.00028	Predicted Protein
**25**	TRINITY_DN4524_c0_g3	0	0	5.98	113.8	426.21	83.05	-9.21	0.00327	Predicted Protein

**Table 4:** Top 25 upregulated genes from copper susceptible samples compared to water controls in *Pinus banksiana*.

Rank	Gene ID	Sus 1	Sus 2	Sus 3	Water 1	Water 2	Water 3	logFC	Adj. P. Value	UniProt Description
**0**	TRINITY_DN2786_c0_g1	670.58	354.91	364.1	0	0	0	13.15	8.45E-06	Predicted Protein
**1**	TRINITY_DN1628_c0_g1	1238.41	1180.01	590.75	0	0.32	0	12.80	2.48E-05	Trypsin inhibitor [Cleaved into: Trypsin inhibitor chain A; Trypsin inhibitor chain B]
**2**	TRINITY_DN258556_c0_g1	248.22	356.86	541.07	0	0	0	12.77	1.88E-05	Predicted Protein
**3**	TRINITY_DN1368_c0_g1	1712.84	2189.53	1629.49	0	1.3	0.07	12.73	2.67E-06	Predicted Protein
**4**	TRINITY_DN5716_c0_g1	2481.86	3248.79	5881.21	0	7.03	0.41	12.53	6.37E-04	Predicted Protein
**5**	TRINITY_DN2832_c0_g1	358.6	494.32	122.28	0	0	0	12.49	3.11E-05	Predicted Protein
**6**	TRINITY_DN5391_c1_g1	496.98	221.21	166.02	0	0	0	12.43	3.94E-05	Predicted Protein
**7**	TRINITY_DN57079_c0_g1	223.25	327.76	184.27	0	0	0	12.22	1.57E-07	Predicted Protein
**8**	TRINITY_DN5965_c1_g1	799.48	842.36	692.63	0.33	0	0	12.12	2.95E-06	Predicted Protein
**9**	TRINITY_DN50999_c1_g1	333.04	156.25	133.24	0	0	0	11.95	1.73E-05	Predicted Protein
**10**	TRINITY_DN55243_c0_g1	115.54	286.47	226.14	0	0	0	11.88	1.05E-05	Predicted Protein
**11**	TRINITY_DN1520_c0_g1	1020.58	623.65	1427.83	0.02	0.65	0	11.86	1.18E-03	Trypsin inhibitor [Cleaved into: Trypsin inhibitor chain A; Trypsin inhibitor chain B]
**12**	TRINITY_DN5795_c0_g1	851.63	854.34	325.97	0	0.84	0	11.83	2.09E-04	Predicted Protein
**13**	TRINITY_DN7061_c1_g1	194.74	291.48	98.46	0	0	0	11.82	2.94E-06	Predicted Protein
**14**	TRINITY_DN8563_c1_g1	131.04	322.9	115.04	0	0	0	11.71	4.77E-06	Predicted Protein
**15**	TRINITY_DN4524_c0_g3	113.8	426.21	83.05	0	0	0	11.63	9.67E-05	Predicted Protein
**16**	TRINITY_DN257933_c1_g1	201.53	169.25	77.24	0	0	0	11.48	4.34E-06	Predicted Protein
**17**	TRINITY_DN3536_c0_g1	122.8	169.34	90.42	0	0	0	11.28	3.13E-07	Predicted Protein
**18**	TRINITY_DN237688_c0_g1	211.18	103.61	72.51	0	0	0	11.25	1.84E-05	Predicted Protein
**19**	TRINITY_DN7685_c0_g1	509.79	347.25	228.34	0	0.41	0	11.24	6.78E-05	Predicted Protein
**20**	TRINITY_DN14732_c0_g1	372.88	97.02	31.35	0	0	0	11.18	1.92E-03	Predicted Protein
**21**	TRINITY_DN12750_c0_g1	166.37	64.69	116.96	0	0	0	11.11	3.61E-05	Predicted Protein
**22**	TRINITY_DN2463_c0_g1	818.33	234.67	289.22	0	0.04	0.11	11.02	2.24E-03	Predicted Protein
**23**	TRINITY_DN3069_c0_g1	313.54	458.61	155.11	0	0.37	0	10.98	6.68E-05	Predicted Protein
**24**	TRINITY_DN2221_c0_g1	664.03	226.26	181.23	0	0.68	0	10.90	1.96E-03	Predicted Protein
**25**	TRINITY_DN3092_c0_g1	661.05	923.49	459.73	0.14	0.35	0	10.88	6.47E-06	Glucan endo-1,3-beta-glucosidase, acidic isoform, EC 3.2.1.39 ((1->3)-beta-glucan endohydrolase, (1->3)-beta-glucanase) (Beta-1,3-endoglucanase)

**Table 5:** Top 25 downregulated genes from copper susceptible samples compared to water controls in *Pinus banksiana*.

Rank	Gene ID	Sus 1	Sus 2	Sus 3	Water 1	Water 2	Water 3	logFC	Adj. P. value	UniProt Description
**0**	TRINITY_DN293_c0_g1	0.02	0	4.27	83.3	131.42	164.86	-11.03	5.8E-05	Delta-selinene-like synthase, chloroplastic, PsTPS-Sell, EC 4.2.3.76
**1**	TRINITY_DN293_c0_g1	0.02	0	4.27	83.3	131.42	164.86	-11.03	5.8E-05	Alpha-humulene synthase, EC 4.2.3.104 (Terpene synthase TPS-Hum, PgTPS-Hum)
**2**	TRINITY_DN293_c0_g1	0.02	0	4.27	83.3	131.42	164.86	-11.03	5.8E-05	Delta-selinene synthase, EC 4.2.3.71, EC 4.2.3.76 (Agfdsel1)
**3**	TRINITY_DN5038_c0_g2	0.21	0.18	1.49	147.03	102.4	230.91	-10.45	2.0E-04	Predicted Protein
**4**	TRINITY_DN1269_c0_g1	0	0	0.13	32.25	13.5	37.33	-10.30	1.4E-04	Predicted Protein
**5**	TRINITY_DN4890_c0_g1	0	0	0	15.59	12.05	25.46	-10.25	7.2E-06	Predicted Protein
**6**	TRINITY_DN2314_c0_g1	0.03	0.05	0.05	40.88	14.56	52.04	-10.14	7.2E-04	Predicted Protein
**7**	TRINITY_DN8038_c0_g1	0.16	0.3	0.98	122.65	81.13	105.38	-10.02	7.8E-05	Probable aquaporin PIP2-8 (Plasma membrane intrinsic protein 2–8, AtPIP2;8) (Plasma membrane intrinsic protein 3b, PIP3b)
**8**	TRINITY_DN26931_c0_g1	1.66	0	0	65.61	45.82	36.39	-9.96	3.1E-04	Probable aquaporin PIP2-8 (Plasma membrane intrinsic protein 2–8, AtPIP2;8) (Plasma membrane intrinsic protein 3b, PIP3b)
**9**	TRINITY_DN10618_c0_g1	0	0	0	17.92	13.75	10	-9.96	9.8E-06	Predicted Protein
**10**	TRINITY_DN4059_c0_g1	0.07	0	0	20.09	12.1	19.58	-9.78	1.7E-05	Predicted Protein
**11**	TRINITY_DN159567_c0_g1	0	0	0	11.89	6.31	15.47	-9.60	2.5E-05	WAT1-related protein At5g07050
**12**	TRINITY_DN34182_c0_g1	0	0	0	11.56	9.9	10.5	-9.59	2.7E-06	Predicted Protein
**13**	TRINITY_DN129749_c0_g1	0	0	0	9.6	9.98	11.12	-9.52	1.5E-06	Predicted Protein
**14**	TRINITY_DN129793_c0_g1	0	0	0	8.28	13.37	9.36	-9.48	1.2E-06	Putative UPF0481 protein At3g02645
**15**	TRINITY_DN2617_c0_g1	0	0	0	12.84	6.76	7.48	-9.34	2.4E-05	Predicted Protein
**16**	TRINITY_DN11362_c0_g1	0	0.76	0.54	39.53	30.88	57.71	-9.33	1.4E-04	Predicted Protein
**17**	TRINITY_DN4176_c0_g1	0.47	0	0.44	61.56	18.42	66.16	-9.31	3.9E-03	Chalcone synthase, EC 2.3.1.74 (Naringenin-chalcone synthase)
**18**	TRINITY_DN2507_c0_g1	0	1.1	0	32.43	13.12	23.67	-9.23	6.4E-04	Predicted Protein
**19**	TRINITY_DN113586_c0_g1	0	0	0	7.25	5.74	13.28	-9.21	1.3E-05	Predicted Protein
**20**	TRINITY_DN1400_c0_g1	0.07	0.06	0.21	24.93	11.67	26.83	-9.20	2.6E-04	Subtilisin-like protease SBT5.6, EC 3.4.21.- (Subtilase subfamily 5 member 6, AtSBT5.6)
**21**	TRINITY_DN26605_c0_g1	0	0	0	6.61	7.11	10.35	-9.13	2.9E-06	Predicted Protein
**22**	TRINITY_DN7878_c0_g1	0.4	1.2	1.38	124.44	78.86	175.53	-9.11	5.2E-05	Predicted Protein
**23**	TRINITY_DN1514_c0_g1	0.27	0.75	1.81	82.92	99.69	154.44	-9.11	4.5E-06	Germin-like protein 8–14 (Germin-like protein 1) (Germin-like protein 5, OsGER5)
**24**	TRINITY_DN21458_c0_g1	0	0	0	8.31	6.63	7.86	-9.11	4.5E-06	Predicted Protein
**25**	TRINITY_DN69830_c0_g4	0.03	0.09	0.04	10.13	7.29	18.59	-9.10	4.0E-05	Predicted Protein

**Table 6:** Identified candidate genes from the top upregulated genes in copper resistant vs copper susceptible *Pinus banksiana*.

Rank	Gene ID	Res 1	Res 2	Res 3	Sus 1	Sus 2	Sus 3	logFC	Adj. P. Value	UniProt Description
80	TRINITY_DN6541_c0_g1	251.91	62.08	39.98	4.26	0.53	3.88	6.93	0.00080	Heavy metal-associated isoprenylated plant protein 20, AtHIP20, AtHIPP20
81	TRINITY_DN6541_c0_g1	251.91	62.08	39.98	4.26	0.53	3.88	6.93	0.00080	Heavy metal-associated isoprenylated plant protein 26, AtHIP26, AtHIPP26 (Farnesylated protein 6, AtFP6)
87	TRINITY_DN55790_c0_g1	6.62	5.2	3.26	0	0.87	0	6.90	0.00072	Pleiotropic drug resistance protein 1 (NtPDR1)

### Biological processes

#### Upregulated genes

**Fig 3:**
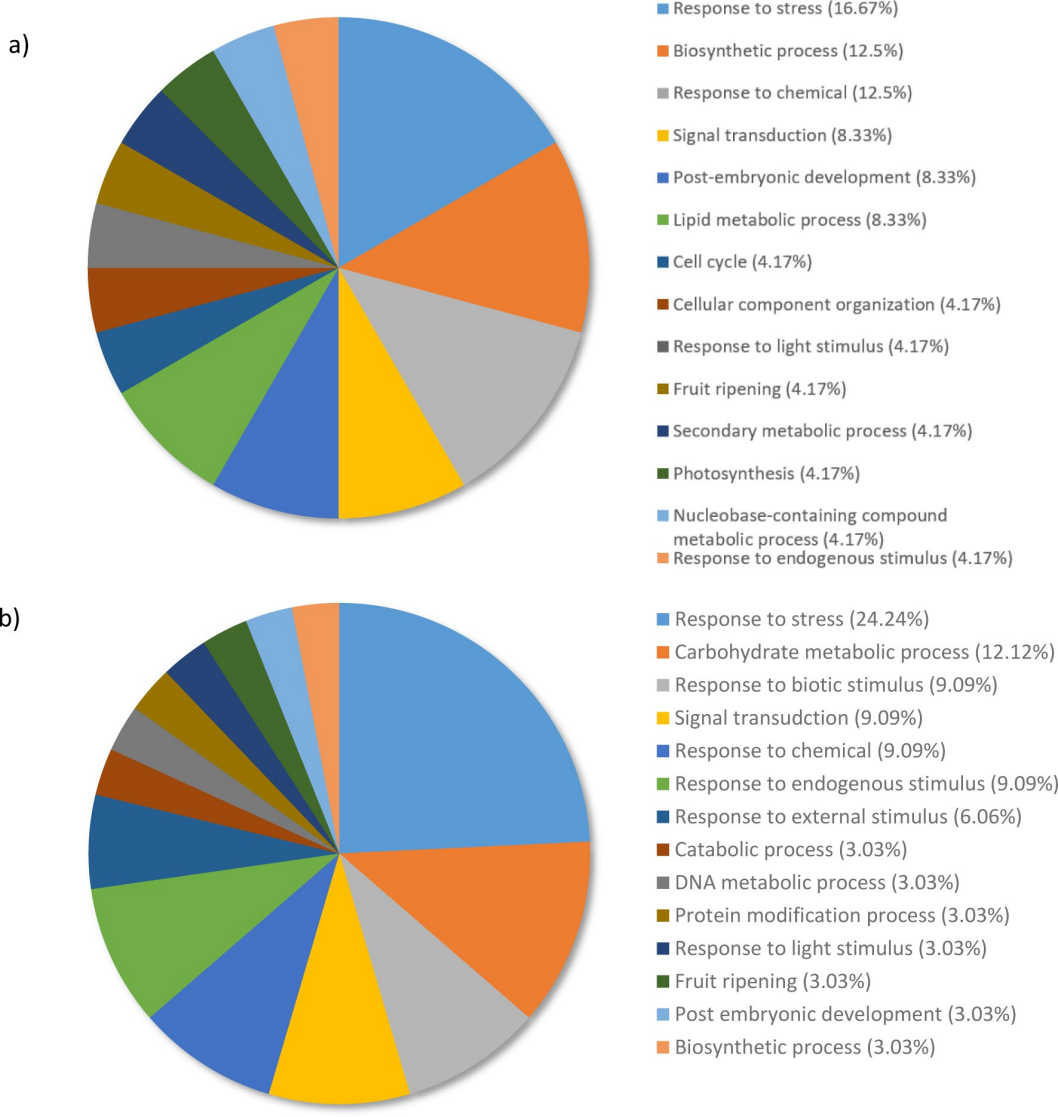
The top 100 most upregulated genes from a) resistant genotypes compared to the susceptible genotypes and b) susceptible genotypes compared to water controls. They were grouped by Gene Ontology terms within the Biological Processes category using Omicsbox/BLAST2GO. Terms with lower than 2% of total gene expression were combined together and assigned the label “other”.

Fig 3 shows the top upregulated genes of RG compared to SG and susceptible compared to water distributed into different terms within the Biological processes category. For this category, the top upregulated genes revealed that the response to stress term (16.67%) comprised the largest proportion of gene expression followed by the biosynthetic process (12.5%), response to chemical (12.5%), signal transduction (8.33%), post-embryonic development (8.33%) and the lipid metabolic process (8.33%) (Fig 3A). These terms represented 66.66% of the expressed genes. Response to stress, response to chemical, response to light stimulus and response to endogenous stimulus share the parent term response to stimulus.

When SG were compared to the water controls for the biological processes category, 63.63% of upregulated genes were distributed to the following subcategories: response to stress (24.24%), carbohydrate metabolic process (12.12%), response to biotic stimulus (9.09%), response to chemical (9.09%), and response to endogenous stimulus (9.09%) (Fig 3B). Five of the top 10 categories fall under the parent term response to stimulus.

### Downregulated genes

**Fig 4:**
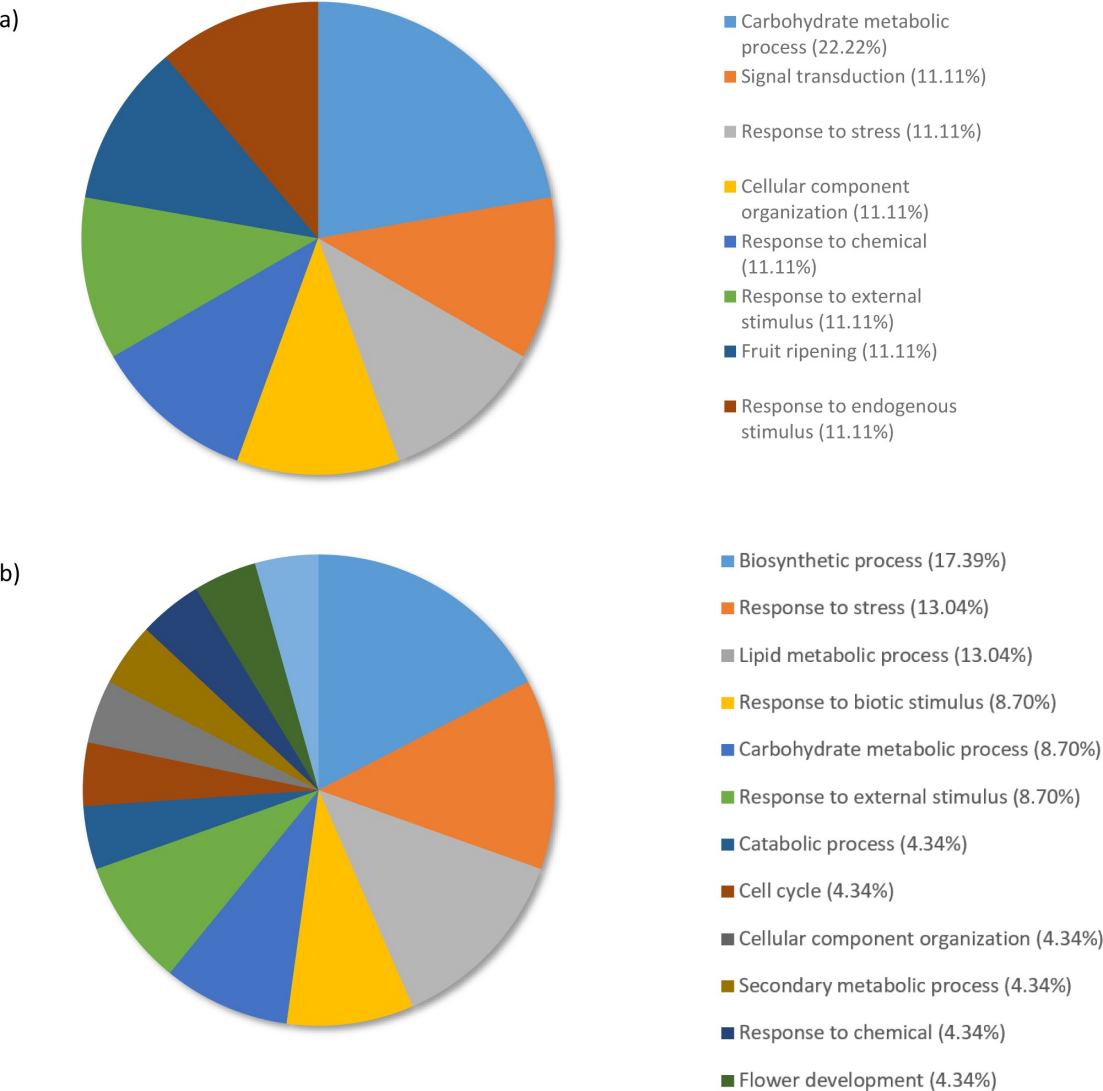
Percentage of the top 100 downregulated transcripts in *Pinus banksiana* from a) resistant compared to the susceptible genotypes and b) susceptible genotypes compared to water controls. They were grouped by Gene Ontology terms within the Biological Processes category using Omicsbox/BLAST2GO. Terms with lower than 2% of total gene expression were combined together and assigned the label “other”.

When RG were compared to the SG, for the biological processes category, 22.22% of genes were distributed to the carbohydrate metabolic process term (Fig 4A). Genes coding for signal transduction, response to stress, cellular component organisation, response to chemical, response to external stimulus, fruit ripening, and response to endogenous stimulus represented each 11,11% of the top downregulated genes (Fig 4A). Response to stress, response to chemicals, response to external stimulus, and response to endogenous stimulus share the parent term response to stimuli.

When SG were compared to the water control, for the Biological processes category, 52.17% of the downregulated genes were allocated to biosynthetic process (17.39%), response to stress (13.04%), lipid metabolic process (13.04%), and response to biotic stimulus (8.70%) (Fig 4B).

### Metabolic function

#### Upregulated genes

**Fig 5:**
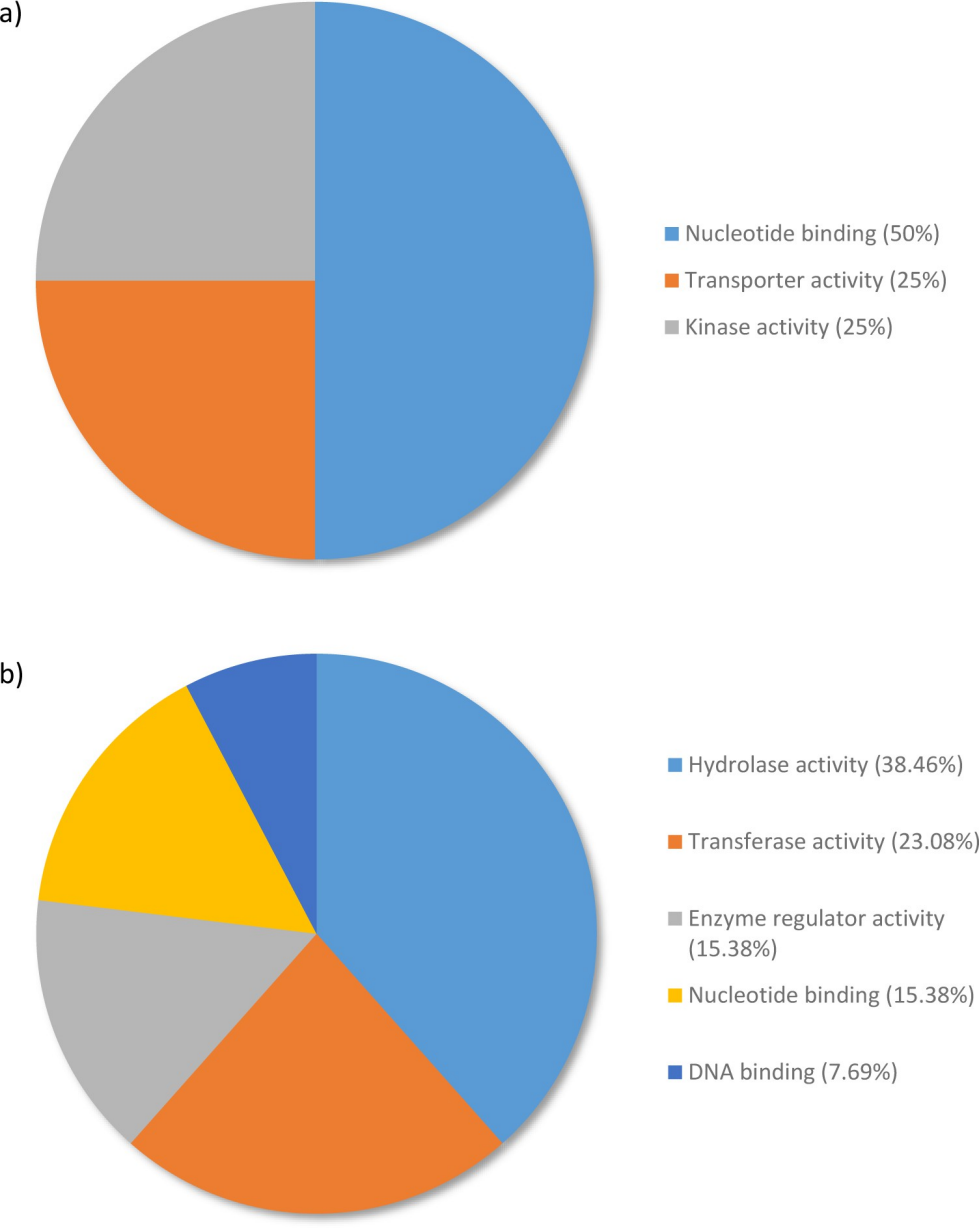
Percentage of the top 100 Upregulated transcripts in *Pinus banksiana* a) resistant genotypes compared to susceptible genotypes and b) susceptible samples compared to water categorized by Molecular Function. They were grouped by Gene Ontology terms within the Molecular Function category using Omicsbox/BLAST2GO. Terms with lower than 2% of total gene expression were combined together and assigned the label “other”.

When RG were compared to SG, analysis of top upregulated genes revealed that 50% of expressed genes were associated with nucleotide binding followed by transporter activity (25%) and kinase activity (25%) (Fig 5A). This pattern changes when SG were compared to water with 61.54% of the top upregulated genes distributed to the hydrolase activity (38.46%) and transferase activity (23.08%) (Fig 5B). Hydrolase activity and transferase activity are categories that fall under the parent category catalytic activity whereas nucleotide binding and DNA binding categories are associated with nucleotide function.

#### Downregulated genes

**Fig 6:**
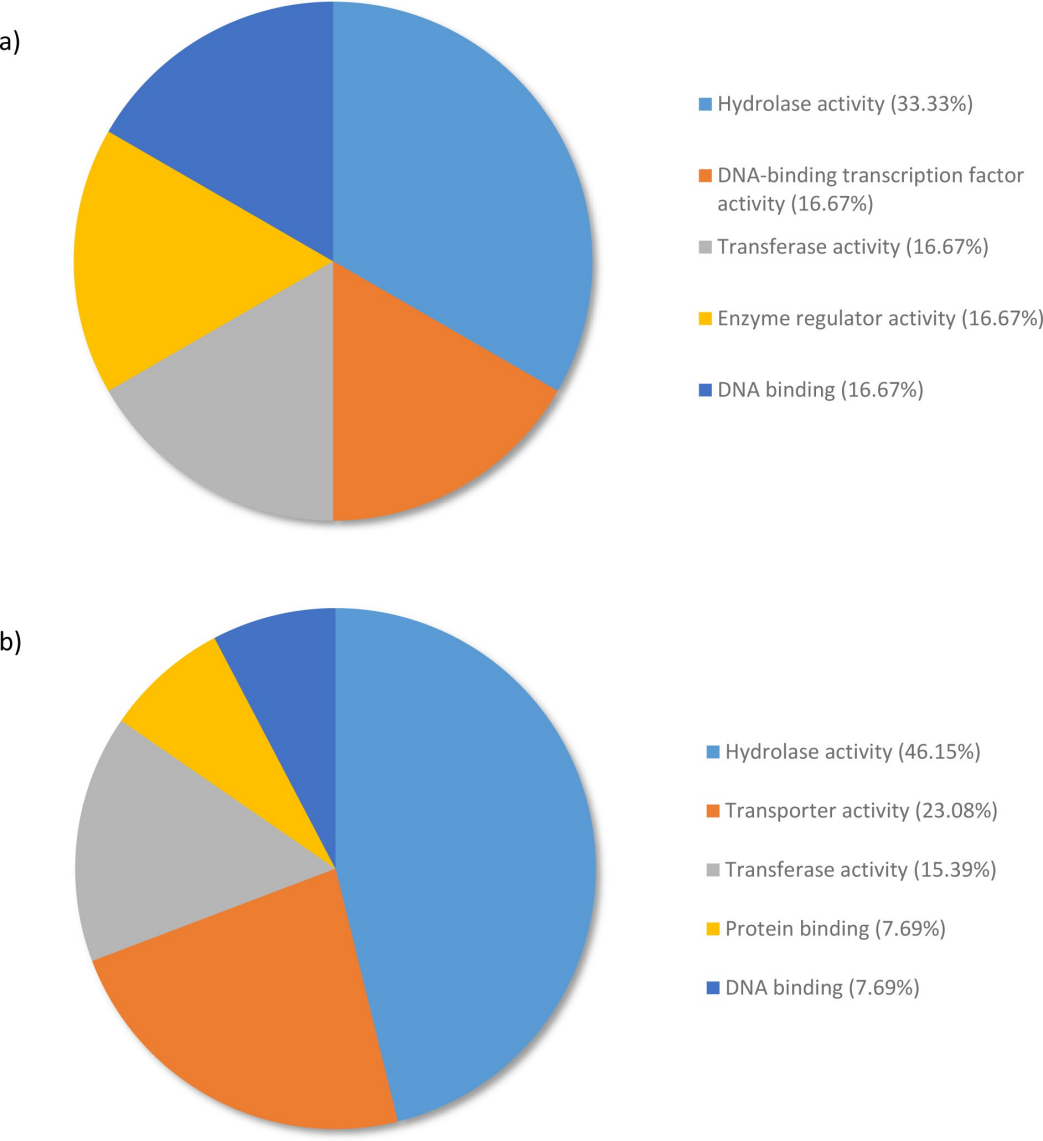
Percentage of the top 100-downregulated transcripts in *Pinus banksiana* a) resistant genotypes compared to susceptible genotypes and b) susceptible samples compared to water categorized by Molecular Function. They were grouped by Gene Ontology terms within the Molecular Function category using Omicsbox/BLAST2GO. Terms with lower than 2% of total gene expression were combined together and assigned the label “other”.

Analysis of downregulated genes revealed that for the metabolic process category, 66.67% of expressed genes were allocated to the terms hydrolase activity (33.33%), DNA-binding transcription factor activity (16.67%), and transferase activity (16.67%) when RG and SG were compared (Fig 6A).

A different pattern of downregulated genes was observed when SG were compared to the water control. In fact, 69.23% of the expressed genes were categorized under hydrolase activity (46.15%) and transporter activity (23.08%) (Fig 6B).

### Cellular compartment

#### Upregulated genes

When RG were compared to SG, for the cellular component category, the membrane term comprised the largest portion of the top upregulated genes (60%) (S1a Fig in S2 File). The thylakoid organelle is also represented in the top upregulated genes. However, 66.67% of upregulated genes were distributed to the extracellular region (41.67%) and nucleus (25%) when SG were compared to water (S1b Fig in S2 File).

#### Downregulated genes

The pattern of downregulated genes showed that for the cellular component category, 50% of expressed genes was associated with the extracellular region followed by the terms membrane (25%) and nucleus (25%) (S2a Fig in S2 File) when RG and SG were compared. Comparison of SG and water revealed that extracellular region (42.11%) and plasma membrane (21.05%) had the highest proportion of the top downregulated genes (S2b Fig in S2 File).

## Discussion

### DGE analysis of copper genotypes

A transcriptome analysis of *Pinus banksiana* treated with excess copper was performed to evaluate differences in global gene expression between different genotypes. Analyzing the transcriptome of resistant and susceptible seedlings can provide valuable insight on the molecular mechanisms associated with copper resistance or susceptibility in *Pinus banksiana*. The high number of DEGs in RG compared to SG at high stringency indicated that copper resistant plants had a significantly different pattern of gene expression compared to copper susceptible plants. Notably, the large proportion of downregulated genes compared to upregulated genes suggests a decline in many cellular processes. In RG compared to water, the very low number of DEGs at a high stringency indicates a similar pattern of gene expression between resistant plants and untreated plants. Several studies have demonstrated the significance of a single transporter or chelator gene on copper resistance and overall growth outcomes [17, 20, 33]. The large deviation in gene expression for SG compared to both RG and the water group suggests that there may be genetic and molecular factors contributing to copper susceptibility. The number of DEGs was also considerably higher in SG compared to water, implying that copper susceptibility was characterized by a large deviation from untreated conditions. This raises the possibility that DEGs in SG were also associated with death coping mechanisms responding to copper induced stress and tissue damage. This pattern of gene expression was also observed in *Populus tremuloides* when exposed to excess nickel [34].

### Identification of candidate genes associated with copper resistance in *Pinus banksiana*

Analysis of the top upregulated genes revealed two promising candidate genes that may be involved in copper resistance mechanisms for *Pinus banksiana*. A candidate gene encoding a heavy metal-associated isoprenylated plant (HIPP) protein was identified. HIPP proteins are metallochaperones that chelate and deliver heavy metal ions to various proteins across cellular compartments [35]. The conserved protein structure of HIPP family proteins is comprised of a cysteine rich heavy metal associated (HMA) domain facilitating the binding of heavy metals and an isoprenylaton site involved in cellular compartment localization and signal transduction [35–37]. Structural variation within the isoprenylated site offers the functional diversity needed to facilitate cellular compartment localization and protein targeting [37, 38]. In yeast, the overexpression of HIPP conferred resistance to copper, cadmium, and zinc [39]. Additionally, overexpression of HIPP in *Arabidopsis thaliana* conferred cadmium resistance [40]. Studies that demonstrated the binding of HIPP to copper, cadmium and zinc in plants corroborates the function of HIPP in the detoxification of heavy metals [38, 41]. The capture of copper ions by cysteine rich chelators is an essential mechanism that prevents excess copper from inhibiting enzymes, causing protein misfolding and generating ROS [42]. Although the specific protein targeted by AtHIPP20 are currently unknown, possible targets of metallochaperone mediated ion delivery include transporters, enzymes or other chaperones [39].

Another identified candidate gene encodes the Pleiotropic drug resistance (PDR) protein, which is an ATP-binding cassette (ABC) transporter [43]. ABC transporters are membrane localized transporters that facilitate the movement of a large diversity of entities via an ATP hydrolysis motif [44, 45]. The conserved protein region of ABC transporters include two nucleotide binding folds associated with ATP hydrolysis and two hydrophobic membrane domains involved in the determination of substrate specificity [44, 46]. The broad substrate specificity of PDR proteins often includes heavy metals, resulting in its involvement in heavy metal homeostasis as it pertains to the cytosol and the region external to the membrane [47]. In addition to heavy metal, PDR is also able to transport phytohormones, antifungal agents, and metabolites with antimicrobial properties [48–50]. In *Arabidopsis thaliana*, an overexpression of AtPDR8 conferred cadmium resistance and reduced total cadmium content by exporting cadmium from the cytosol to the apoplast [47]. Similarly, upregulated AtPDR12 expression increased lead export from the cytosol which subsequently decreased lead content and contributed to lead resistance [51]. A possible role of PDR in copper detoxification may involve utilizing a similar efflux mechanism to export copper away from the cytosol and plasma membrane. The suppression of PDR in certain species treated with cadmium or lead resulted in severe growth defects, establishing PDR as a crucial component in heavy metal detoxification [51, 52]. Hormones such as Jasmonic acid and ABA induced the upregulation of Ospdr9, suggesting that PDR may also play a secondary role in mediating the general stress response [49, 53]. Further research is needed to describe how PDR contributes to copper detoxification in *Pinus banksiana*.

It should be pointed out that the two putative copper resistance genes encoding for HIPP and PDR proteins were not identified in SG and water-treated genotypes. It is likely that these genes are involved in copper resistance in *P*. *banksiana* since all the tissues collected from the SG were healthy. The effects of observed damages to SG on gene expression in healthy parts of the plants are unknown and cannot be completely discarded. A recent study shows that HIPP gene family plays a crucial role in cadmium resistance and accumulation in woody plant species such as the tea plant (*Camellia sinensis* L.) [54]. This study opens the door to further investigations to validate the role of HIPP and PDR in *P*. *banksiana* resistance to copper.

### GO annotation of the top 25 upregulated genes between the resistant genotype and the susceptible genotype

Many of the top upregulated genes in RG compared to SG were involved in the stress response and may contribute to copper tolerance. The gene encoding terpene synthase (TPS) was among the top upregulated genes. TPS synthesizes 1,8-cineole and other terpenoids that partake in a variety of defense functions such as thermoregulation, resin assisted wound sealing, and plant to plant signalling [55–57]. In response to heavy metals, terpenoids may scavenge ROS, mainly exerting its protective effect on membranes [58]. Terpenoids can also enhance the stability and rigidity of the chloroplast membrane by increasing the hydrophobic bonding between lipids [59, 60]. Enhancing the stability of membranes is a protective mechanism for resisting ROS mediated damage and lipid peroxidation caused by copper toxicity [61, 62].

A gene encoding a Fatty acyl-CoA reductase (FAR) was also identified among the top upregulated genes. FAR catalyzes the synthesis of fatty alcohols from fatty acyl-coA and serves an integral part in the acyl-reduction pathway [63]. Fatty alcohols are used as components for larger extracellular lipid compounds such as cuticular wax and suberin in response to heavy metals [64, 65]. These compounds (cuticular wax and suberin) form a hydrophobic barrier that blocks and protects cells against copper induced water loss when secreted onto the surface of leaves [66, 67]. The negative regulation of water transpiration is crucial to counteract water loss and drought which could induce further tissue damage [67].

Genes encoding Early light-induced proteins (ELIP) were also identified. ELIPs are photoactive proteins that regulate chloroplast content, counteract the photoinhibition of photosystem II, and safeguards photosynthesis machinery [68, 69]. Copper stress decreases chloroplast concentration, diminishes thylakoid membranes and replaces the iron cofactor in plastoquinone QA of photosystem II [9]. Notably, the inhibition of plastoquinone QA results in a reduction of electron transfer and subsequent light absorption [8, 9]. The collective effect of copper on chloroplast function may result in photodamage and ROS mediated damage [70, 71]. Upregulation of ELIP may potentially serve a photoprotective role to preserve chloroplast function and light absorption, although the exact mechanism of action has yet to be described [69].

### GO annotation of the top 25 downregulated genes between the resistant genotype and the susceptible genotype

Several top downregulated genes encode enzymes involved in carbohydrate metabolism. An identified gene encodes polygalactuornase (pectinase), which facilitates the hydrolysis of the alpha-1,4 glycosidic bonds present in polygalacturnoan (pectin) [72]. Pectin is an essential component of the cell wall and is responsible for cell to cell adhesion [73]. Pectinase downregulation could potentially contribute to copper tolerance by preserving pectin content, thereby maintaining the integrity of the cell wall. It has been reported that downregulation of pectinase improved tolerance to multiple stressors by decreasing cell expansion, cell separation and increasing cell density [72, 74, 75]. Several genes that encode for beta glucosidase which is an enzyme that facilitates the conversion of cellobiose to glucose have been identified [76]. Beta glucosidase is an integral component of cellulose breakdown and was found to be expressed in the cell wall of some plants [77, 78]. Cellulose is the most abundant component of the cell wall, cellulose and it provides the tensile and turgor pressure required to maintain structural integrity [79, 80]. Downregulation of beta glucosidase upregulates cellulose production and also contributes to the maintenance of the cell wall in response to various stressors [81, 82]. The cell wall plays a crucial role in copper detoxification by acting as a site of sequestration and heavy metal distribution [83, 84]. The role of pectinase and beta glucosidase in maintaining the integrity of the cell wall is supported by the upregulation of the PDR transporter candidate gene, which has been previously shown to transport cadmium from the cytosol to the apoplast [47].

A gene encoding a trypsin inhibitor was identified in the top downregulated genes. Trypsin inhibitors mitigate the activity of serine proteases and prevent the breakdown of associated proteins [85]. Protein damage and misfolding caused by heavy metal binding and ROS interaction induces the production of serine proteases [86, 87]. Downregulation of trypsin inhibitors suggests that higher levels of serine protease activity were needed to breakdown damage or misfolded proteins thereby improving cell viability. Other studies reported variation in trypsin inhibitor activity in response to excess copper, suggesting that the activity is dependent on the extent of protein damage [88, 89].

### GO annotation of the top 25 upregulated genes between the susceptible genotype and the control

In addition to stress related mechanisms triggered by excess copper, the examination of upregulated genes observed when SG and water were compared could reveal genes associated with copper susceptibility or plant cell death and necrosis. Genes encoding the trypsin inhibitor, were among the top upregulated genes. They were previously found to be downregulated in RG when compared to SG. The upregulation of trypsin inhibitors in SG may indicate a larger amount of serine protease activity. Increased ROS production and undesired copper binding cause protein misfolding and damage which can elicit serine protease activity [86, 87]. Overexpression of serine protease may damage plant tissue and cause a further reduction in protein content [90]. The upregulation of trypsin inhibitors could therefore be a protective strategy to conserve protein content and delay senescence [91, 92]. Upregulation of trypsin inhibitors in other plants has also been reported in response to other stressors such as drought and fungal infection [93, 94].

In contrast to gene expression observed when RG compared to SG, many of the top upregulated genes encoded beta glucosidase. The upregulation of beta glucosidase increases the conversion of cellobiose and other sugars to glucose, implying a metabolic related function. An adverse side effect of upregulated beta glucosidase is a decrease in cellulose content which may compromise the strength and integrity of the cell wall [95]. Some studies demonstrated that beta glucosidase may play a possible role in the stress response by regulating ABA levels [96–98]. Other studies reported a correlation between beta glucosidase overexpression and the production of antioxidant flavonols which may also contribute to stress alleviation [99].

### GO annotation of the top 25 downregulated genes between the susceptible genotype and the control

Among the top downregulated genes in SG compared to the control were genes encoding the probable aquaporin proteins PIP2-8. Aquaporins are membrane bound channels that serve as an important point of entry for water, nutrients, and heavy metals [100]. The broad specificity of Aquaporins provides a potential point of regulation to control the transport of heavy metals. Downregulation of aquaporins could also be a response to increased transpiration and water loss caused by heavy metals [101].

A gene encoding the WALLS ARE THIN1 (WAT1) protein was also identified among the top downregulated genes. WAT1 is an auxin transporter that is localized to the vacuole, facilitating the movement of auxin to the cytoplasm [102]. Excess copper can deregulate auxin homeostasis and distribution which can negatively impact various aspects of plant development [103, 104]. In particular, the deregulation of auxin can affect cell division, cell elongation, leaf morphogenesis, and hormone crosstalk, [103, 105, 106]. In response to copper stress, the downregulation of WAT1 could be a strategy to safeguard growth by altering the transport and intracellular distribution of auxin. However, the response of WAT1 to copper specifically is not fully understood. Downregulation of WAT1 may also regulate salicylic acid synthesis which coordinates the stress response [107, 108].

## Conclusion

A comprehensive transcriptome analysis was conducted on copper treated *Pinus banksiana* to understand the genetic response of different genotypes to copper. There were 19,789 DEGs between RG and SG at a high stringency, indicating significant differences in gene expression between resistant and susceptible plants. The low number of DEGs when RG were compared to water control indicated a similar pattern of gene expression. SG had a large number of DEGs compared to both RG and the control, suggesting that SG had a different set of coping mechanisms from the aforementioned groups. It is also possible that genes associated with plant death might interfere with the expression of some genes even if the tissues analyzed were undamaged.

Gene Ontology of the top upregulated genes in RG compared to SG showed that the response to stress had the highest proportion of expressed genes. The carbohydrate metabolic process term had the highest percentage of downregulated genes. *AtHIPP20* and *AtHIPP26* encoding a metallochaperone along with *NtPDR1* encoding an ATP binding cassette transporter, were candidate genes associated with copper resistance in *P*. *banksiana*. Other identified top upregulated genes were associated with the coordination of the stress response and included TPS, AtFAR2 and ELIP1. Top downregulated genes included polygalacturonase and beta glucosidase that may play a role in the strengthening of the cell wall and the sequestration of copper ions. This study demonstrated the strong utility of transcriptome analysis for elucidating the genetic response of plants and associated genotypes to copper stress. The identified candidate genes should be further researched to evaluate potential applications in various industries.

## Supporting information

S1 File (DOCX)

S2 File (DOCX)
