# Supplementary material for: Gene expression profiling of Jack Pine (Pinus banksiana) under copper stress: Identification of genes associated with copper resistance
Source: PLoS One. 2024 Mar 7;19(3):e0296027. doi: 10.1371/journal.pone.0296027 (PMC10919686; doi:10.1371/journal.pone.0296027)
Supplement: S1 File — (DOCX) [file pone.0296027.s001.docx]

**Table 1S**. Damage rating scale and plant classification based on reaction to nickel and copper treatments.

| **% of Leaf area with chlorosis/necrosis** | **Damage Rating** | **Genotype Classification** |
| --- | --- | --- |
| 0-10 | 1 |  |
| 10-20 | 2 | Resistant (RG) |
| 20-30 | 3 |  |
| 30-40 | 4 |  |
| 40-50 | 5 | Moderately Susceptible (MSG) |
| 50-60 | 6 |  |
|  |  |  |
| 60-70 | 7 |  |
| 70-80 | 8 | Susceptible (SG) |
| > 80 | 9 |  |

**Table 2S.** Top 50 upregulated genes from copper resistant samples compared to copper susceptible samples in *Pinus banksiana*

| Rank | Gene ID | Res 1 | Res 2 | Res 3 | Sus 1 | Sus 2 | Sus 3 | logFC | Adj. P. Value | UniProt Description |
| --- | --- | --- | --- | --- | --- | --- | --- | --- | --- | --- |
| 0 | TRINITY_DN35689_c0_g1 | 12.05 | 7.41 | 11.07 | 0 | 0 | 0 | 9.21 | 0.00002 | Predicted Protein |
| 1 | TRINITY_DN10618_c0_g1 | 17.42 | 2.72 | 9.6 | 0 | 0 | 0 | 8.82 | 0.00181 | Predicted Protein |
| 2 | TRINITY_DN91621_c0_g2 | 13.93 | 4.82 | 5.68 | 0 | 0 | 0 | 8.78 | 0.00025 | Predicted Protein |
| 3 | TRINITY_DN199894_c0_g2 | 9.44 | 9.02 | 1.99 | 0 | 0 | 0 | 8.45 | 0.00117 | Predicted Protein |
| 4 | TRINITY_DN28042_c0_g3 | 4.91 | 8.95 | 2.74 | 0 | 0 | 0 | 8.26 | 0.00022 | Cytochrome P450 750A1, EC 1.14.-.- (Cytochrome P450 CYPC) |
| 5 | TRINITY_DN7900_c0_g1 | 6 | 6.27 | 3.22 | 0 | 0 | 0 | 8.25 | 0.00007 | Predicted Protein |
| 6 | TRINITY_DN2617_c0_g1 | 8.29 | 4.5 | 3.27 | 0 | 0 | 0 | 8.24 | 0.00017 | Predicted Protein |
| 7 | TRINITY_DN20922_c0_g1 | 3.17 | 6.13 | 4.05 | 0 | 0 | 0 | 8.02 | 0.00004 | Predicted Protein |
| 8 | TRINITY_DN236262_c0_g1 | 11.08 | 1.85 | 3.41 | 0 | 0 | 0 | 7.96 | 0.00197 | Predicted Protein |
| 9 | TRINITY_DN95006_c0_g1 | 6.22 | 4.28 | 1.99 | 0 | 0 | 0 | 7.87 | 0.00033 | Predicted Protein |
| 10 | TRINITY_DN219929_c1_g1 | 2.74 | 4.62 | 4.69 | 0 | 0 | 0 | 7.86 | 0.00003 | Predicted Protein |
| 11 | TRINITY_DN4529_c0_g1 | 3 | 7.73 | 1.66 | 0 | 0 | 0 | 7.73 | 0.00069 | 1,8-cineole synthase, chloroplastic, EC 4.2.3.108 (Terpene synthase TPS-Cin, PgTPS-Cin) |
| 12 | TRINITY_DN13781_c0_g3 | 16.16 | 15.27 | 4.31 | 0 | 0 | 1.35 | 7.73 | 0.00318 | Predicted Protein |
| 13 | TRINITY_DN216_c0_g1 | 58.07 | 22.76 | 12.46 | 0.07 | 0.49 | 0.97 | 7.72 | 0.00440 | Fatty acyl-CoA reductase 2, chloroplastic, AtFAR2, EC 1.2.1.84 (Fatty acid reductase 2) (Male sterility protein 2) |
| 14 | TRINITY_DN9994_c0_g1 | 8.13 | 1.94 | 2.38 | 0 | 0 | 0 | 7.67 | 0.00114 | Predicted Protein |
| 15 | TRINITY_DN5226_c2_g1 | 3.74 | 2.06 | 5.32 | 0 | 0 | 0 | 7.66 | 0.00014 | Predicted Protein |
| 16 | TRINITY_DN57458_c1_g1 | 2.43 | 3.07 | 4.49 | 0 | 0 | 0 | 7.58 | 0.00004 | Predicted Protein |
| 17 | TRINITY_DN3869_c0_g1 | 3.23 | 4.31 | 2.12 | 0 | 0 | 0 | 7.58 | 0.00009 | Predicted Protein |
| 18 | TRINITY_DN47098_c0_g1 | 7.77 | 2.08 | 1.81 | 0 | 0 | 0 | 7.58 | 0.00131 | Predicted Protein |
| 19 | TRINITY_DN19214_c1_g2 | 3.44 | 3.21 | 2.82 | 0 | 0 | 0 | 7.57 | 0.00004 | Predicted Protein |
| 20 | TRINITY_DN40558_c0_g2 | 1.22 | 7.95 | 3.16 | 0 | 0 | 0 | 7.57 | 0.00127 | Predicted Protein |
| 21 | TRINITY_DN18490_c0_g1 | 2.06 | 7.43 | 1.88 | 0 | 0 | 0 | 7.57 | 0.00066 | Predicted Protein |
| 22 | TRINITY_DN9649_c1_g3 | 0.84 | 8.97 | 3.91 | 0 | 0 | 0 | 7.54 | 0.00382 | Predicted Protein |
| 23 | TRINITY_DN148688_c0_g2 | 6.51 | 3.72 | 1.03 | 0 | 0 | 0 | 7.54 | 0.00203 | Predicted Protein |
| 24 | TRINITY_DN100_c0_g2 | 7.29 | 15.7 | 4.96 | 0 | 0.41 | 0.08 | 7.53 | 0.00072 | Predicted Protein |
| 25 | TRINITY_DN178176_c0_g1 | 3.36 | 4.4 | 1.77 | 0 | 0 | 0 | 7.53 | 0.00017 | Predicted Protein |
| 26 | TRINITY_DN3659_c0_g1 | 8.49 | 2.81 | 0.93 | 0 | 0 | 0 | 7.50 | 0.00393 | Predicted Protein |
| 27 | TRINITY_DN10188_c2_g1 | 2.92 | 2.4 | 3.97 | 0 | 0 | 0 | 7.49 | 0.00005 | Predicted Protein |
| 28 | TRINITY_DN12221_c2_g1 | 6.18 | 0.9 | 5.2 | 0 | 0 | 0 | 7.47 | 0.00372 | Predicted Protein |
| 29 | TRINITY_DN20798_c1_g1 | 1.28 | 5.26 | 3.85 | 0 | 0 | 0 | 7.47 | 0.00044 | Predicted Protein |
| 30 | TRINITY_DN18848_c2_g1 | 1.01 | 9.32 | 2.52 | 0 | 0 | 0 | 7.47 | 0.00293 | Predicted Protein |
| 31 | TRINITY_DN16610_c0_g1 | 1.37 | 5.49 | 3.34 | 0 | 0 | 0 | 7.46 | 0.00038 | Predicted Protein |
| 32 | TRINITY_DN22750_c0_g1 | 4.64 | 1.9 | 2.79 | 0 | 0 | 0 | 7.45 | 0.00023 | Predicted Protein |
| 33 | TRINITY_DN21758_c0_g1 | 5.12 | 3.05 | 1.37 | 0 | 0 | 0 | 7.44 | 0.00060 | Predicted Protein |
| 34 | TRINITY_DN191914_c0_g1 | 1.21 | 2.46 | 9.54 | 0 | 0 | 0 | 7.44 | 0.00172 | Predicted Protein |
| 35 | TRINITY_DN61037_c1_g1 | 4.43 | 3.34 | 1.34 | 0 | 0 | 0 | 7.41 | 0.00048 | Predicted Protein |
| 36 | TRINITY_DN51306_c0_g1 | 32.05 | 61.43 | 35.69 | 0.19 | 0.97 | 7.2 | 7.39 | 0.00007 | Predicted Protein |
| 37 | TRINITY_DN107831_c0_g1 | 3.04 | 2.65 | 2.56 | 0 | 0 | 0 | 7.38 | 0.00005 | Predicted Protein |
| 38 | TRINITY_DN94182_c0_g3 | 4.2 | 4.9 | 0.81 | 0 | 0 | 0 | 7.36 | 0.00247 | Predicted Protein |
| 39 | TRINITY_DN6981_c0_g1 | 5.02 | 1.85 | 2.01 | 0 | 0 | 0 | 7.34 | 0.00043 | Predicted Protein |
| 40 | TRINITY_DN11710_c0_g2 | 150.88 | 92.2 | 20.39 | 1.25 | 0.47 | 3.43 | 7.33 | 0.00128 | Predicted Protein |
| 41 | TRINITY_DN45928_c0_g1 | 2.12 | 1.09 | 8.85 | 0 | 0 | 0 | 7.27 | 0.00239 | Predicted Protein |
| 42 | TRINITY_DN18761_c1_g1 | 11.47 | 2.81 | 7.04 | 0 | 0.78 | 0 | 7.27 | 0.00242 | Predicted Protein |
| 43 | TRINITY_DN2127_c0_g1 | 124.49 | 62.29 | 28.73 | 1.17 | 0.35 | 5.16 | 7.23 | 0.00034 | Early light-induced protein 1, chloroplastic |
| 44 | TRINITY_DN267012_c0_g1 | 2.7 | 3.15 | 1.72 | 0 | 0 | 0 | 7.23 | 0.00011 | Predicted Protein |
| 45 | TRINITY_DN6038_c8_g1 | 4.83 | 1.74 | 1.73 | 0 | 0 | 0 | 7.22 | 0.00056 | Predicted Protein |
| 46 | TRINITY_DN25814_c0_g1 | 1.52 | 1.55 | 7.34 | 0 | 0 | 0 | 7.21 | 0.00101 | Predicted Protein |
| 47 | TRINITY_DN1258_c1_g1 | 8.13 | 6.28 | 5.36 | 0 | 0 | 0.94 | 7.20 | 0.00047 | Predicted Protein |
| 48 | TRINITY_DN107808_c1_g2 | 5.12 | 5.07 | 3.74 | 0.03 | 0.02 | 0.29 | 7.19 | 0.00019 | Predicted Protein |
| 49 | TRINITY_DN18729_c0_g1 | 1.71 | 4.11 | 1.89 | 0 | 0 | 0 | 7.19 | 0.00018 | Predicted Protein |
| 50 | TRINITY_DN870_c0_g2 | 1.27 | 3.87 | 2.91 | 0 | 0 | 0 | 7.18 | 0.00024 | UDP-glycosyltransferase 75C1, Abscisic acid beta-glucosyltransferase, Indole-3-acetate beta-glucosyltransferase, SlUGT75C1, EC 2.4.1.121, EC 2.4.1.263 |

**Table 3S.** Top 50 downregulated genes from copper resistant samples compared to copper susceptible samples in *Pinus banksiana*

| Rank | Gene ID | Res 1 | Res 2 | Res 3 | Sus 1 | Sus 2 | Sus 3 | logFC | Adj. P. Value | UniProt Description |
| --- | --- | --- | --- | --- | --- | --- | --- | --- | --- | --- |
| 0 | TRINITY_DN3519_c0_g1 | 0 | 0 | 0 | 191.35 | 54.37 | 115.22 | -11.34 | 0.00021 | Predicted Protein |
| 1 | TRINITY_DN43547_c0_g1 | 0 | 0 | 0 | 162.58 | 38.33 | 61.13 | -10.81 | 0.00035 | Predicted Protein |
| 2 | TRINITY_DN2824_c0_g1 | 0 | 0.03 | 0 | 90.93 | 154.91 | 84.36 | -10.53 | 0.00000 | Polygalacturonase, PG, EC 3.2.1.15 (Pectinase) |
| 3 | TRINITY_DN2824_c0_g1 | 0 | 0.03 | 0 | 90.93 | 154.91 | 84.36 | -10.53 | 0.00000 | Probable polygalacturonase At1g80170, PG, EC 3.2.1.15 (Pectinase At1g80170) |
| 4 | TRINITY_DN1315_c0_g1 | 0.43 | 0.06 | 0.31 | 763.2 | 1448.5 | 490.5 | -10.28 | 0.00001 | Beta-glucosidase 12, EC 3.2.1.21 |
| 5 | TRINITY_DN1315_c0_g1 | 0.43 | 0.06 | 0.31 | 763.2 | 1448.5 | 490.5 | -10.28 | 0.00001 | Furcatin hydrolase, FH, EC 3.2.1.161 |
| 6 | TRINITY_DN1315_c0_g1 | 0.43 | 0.06 | 0.31 | 763.2 | 1448.5 | 490.5 | -10.28 | 0.00001 | Non-cyanogenic beta-glucosidase, EC 3.2.1.21 |
| 7 | TRINITY_DN1315_c0_g1 | 0.43 | 0.06 | 0.31 | 763.2 | 1448.5 | 490.5 | -10.28 | 0.00001 | Beta-glucosidase 27, Os8bglu27, EC 3.2.1.21 |
| 8 | TRINITY_DN1315_c0_g1 | 0.43 | 0.06 | 0.31 | 763.2 | 1448.5 | 490.5 | -10.28 | 0.00001 | Beta-glucosidase 11, Os4bglu11, EC 3.2.1.21 |
| 9 | TRINITY_DN1315_c0_g1 | 0.43 | 0.06 | 0.31 | 763.2 | 1448.5 | 490.5 | -10.28 | 0.00001 | Beta-glucosidase 24, Os6bglu24, EC 3.2.1.21 |
| 10 | TRINITY_DN1315_c0_g1 | 0.43 | 0.06 | 0.31 | 763.2 | 1448.5 | 490.5 | -10.28 | 0.00001 | Beta-glucosidase 13, Os4bglu13, EC 3.2.1.21 |
| 11 | TRINITY_DN67935_c0_g1 | 0 | 0 | 0 | 16.39 | 97.3 | 73.99 | -10.13 | 0.00080 | Predicted Protein |
| 12 | TRINITY_DN702_c0_g1 | 0 | 0.03 | 0 | 59.4 | 116.45 | 27.95 | -9.93 | 0.00006 | Cytochrome P450 71AU50, EC 1.14.-.- |
| 13 | TRINITY_DN702_c0_g1 | 0 | 0.03 | 0 | 59.4 | 116.45 | 27.95 | -9.93 | 0.00006 | Cytochrome P450 750A1, EC 1.14.-.- (Cytochrome P450 CYPC) |
| 14 | TRINITY_DN2358_c0_g1 | 0 | 0 | 0 | 81.45 | 89.01 | 7.38 | -9.91 | 0.00287 | Predicted Protein |
| 15 | TRINITY_DN31159_c0_g1 | 0 | 0 | 0 | 32.73 | 41.22 | 41.42 | -9.83 | 0.00001 | Predicted Protein |
| 16 | TRINITY_DN157611_c0_g2 | 0 | 0 | 0 | 22.19 | 59.12 | 26.75 | -9.60 | 0.00002 | Predicted Protein |
| 17 | TRINITY_DN10725_c0_g1 | 0 | 0 | 0 | 29.76 | 70.11 | 14.01 | -9.55 | 0.00010 | Predicted Protein |
| 18 | TRINITY_DN30360_c0_g2 | 0 | 0 | 0 | 98.39 | 13.66 | 18.36 | -9.53 | 0.00190 | Predicted Protein |
| 19 | TRINITY_DN251401_c0_g1 | 0 | 0 | 0 | 93.47 | 11.8 | 22.49 | -9.52 | 0.00209 | Predicted Protein |
| 20 | TRINITY_DN27632_c0_g2 | 0 | 0 | 0 | 8.22 | 77.05 | 47.39 | -9.45 | 0.00238 | Predicted Protein |
| 21 | TRINITY_DN10160_c0_g1 | 0 | 0 | 0 | 14.16 | 73.48 | 23.65 | -9.41 | 0.00024 | Predicted Protein |
| 22 | TRINITY_DN1453_c1_g4 | 0 | 0 | 0 | 36.74 | 50.95 | 10.28 | -9.37 | 0.00016 | Predicted Protein |
| 23 | TRINITY_DN57079_c0_g1 | 6.63 | 0 | 0 | 223.25 | 327.76 | 184.27 | -9.36 | 0.00049 | Predicted Protein |
| 24 | TRINITY_DN3979_c0_g1 | 0.05 | 0.01 | 0 | 52.76 | 91.37 | 16.98 | -9.28 | 0.00028 | Predicted Protein |
| 25 | TRINITY_DN4524_c0_g3 | 0 | 0 | 5.98 | 113.8 | 426.21 | 83.05 | -9.21 | 0.00327 | Predicted Protein |
| 26 | TRINITY_DN34759_c0_g1 | 6.79 | 0 | 0 | 322.78 | 155.49 | 180.5 | -9.18 | 0.00153 | Predicted Protein |
| 27 | TRINITY_DN73957_c1_g1 | 0 | 0 | 0 | 11.64 | 28.85 | 41.17 | -9.11 | 0.00023 | Predicted Protein |
| 28 | TRINITY_DN75419_c0_g1 | 0.25 | 0 | 0 | 62.72 | 75.65 | 55.6 | -9.08 | 0.00003 | Predicted Protein |
| 29 | TRINITY_DN9012_c0_g1 | 0 | 0 | 0 | 22.31 | 72.56 | 6.41 | -9.07 | 0.00112 | Predicted Protein |
| 30 | TRINITY_DN1628_c0_g1 | 7.75 | 0.43 | 0 | 1238.41 | 1180.01 | 590.75 | -9.07 | 0.00011 | Trypsin inhibitor [Cleaved into: Trypsin inhibitor chain A; Trypsin inhibitor chain B ] |
| 31 | TRINITY_DN3979_c1_g1 | 0 | 0 | 0 | 47.13 | 42.56 | 4.42 | -9.05 | 0.00236 | Predicted Protein |
| 32 | TRINITY_DN157611_c0_g3 | 0 | 0 | 0 | 17.21 | 32.42 | 19.18 | -9.03 | 0.00001 | Predicted Protein |
| 33 | TRINITY_DN15815_c1_g1 | 0 | 0 | 0 | 6.21 | 49.45 | 40.59 | -9.03 | 0.00232 | Predicted Protein |
| 34 | TRINITY_DN95424_c0_g1 | 0 | 0 | 0 | 11.51 | 54.25 | 15.08 | -8.96 | 0.00022 | Predicted Protein |
| 35 | TRINITY_DN7685_c0_g1 | 0.35 | 0.17 | 0.18 | 509.79 | 347.25 | 228.34 | -8.94 | 0.00001 | Predicted Protein |
| 36 | TRINITY_DN1456_c0_g1 | 1.37 | 0 | 1.34 | 512.91 | 233.93 | 218.11 | -8.92 | 0.00030 | Predicted Protein |
| 37 | TRINITY_DN71807_c0_g2 | 0 | 0 | 0 | 22.55 | 16.31 | 22.17 | -8.92 | 0.00003 | Predicted Protein |
| 38 | TRINITY_DN4477_c1_g1 | 0.88 | 0 | 0 | 81.97 | 139.91 | 53.7 | -8.90 | 0.00024 | Predicted Protein |
| 39 | TRINITY_DN7520_c2_g1 | 0 | 0 | 0 | 9.41 | 27.2 | 33.69 | -8.88 | 0.00027 | Predicted Protein |
| 40 | TRINITY_DN4184_c0_g1 | 0.18 | 0 | 0 | 46.12 | 92.42 | 26.45 | -8.83 | 0.00016 | Predicted Protein |
| 41 | TRINITY_DN7066_c0_g1 | 0 | 0 | 0 | 20.01 | 31.33 | 9.88 | -8.80 | 0.00004 | Predicted Protein |
| 42 | TRINITY_DN933_c0_g1 | 1.8 | 0 | 0.74 | 315.27 | 351.67 | 163.92 | -8.78 | 0.00017 | Predicted Protein |
| 43 | TRINITY_DN1728_c0_g1 | 0 | 0 | 0 | 11.2 | 40.87 | 13.71 | -8.77 | 0.00010 | Predicted Protein |
| 44 | TRINITY_DN58476_c0_g1 | 0 | 0 | 0.69 | 19.97 | 131.68 | 83.6 | -8.71 | 0.00446 | Predicted Protein |
| 45 | TRINITY_DN106984_c0_g1 | 0 | 0 | 0 | 13.93 | 11.45 | 34.39 | -8.69 | 0.00029 | Predicted Protein |
| 46 | TRINITY_DN16651_c1_g1 | 0 | 0 | 0 | 5.99 | 54.4 | 17.47 | -8.68 | 0.00142 | Predicted Protein |
| 47 | TRINITY_DN94859_c1_g2 | 0 | 0 | 0 | 15.4 | 19.32 | 16.7 | -8.67 | 0.00001 | Predicted Protein |
| 48 | TRINITY_DN53823_c0_g1 | 0 | 0 | 0 | 24.5 | 12.76 | 14.3 | -8.66 | 0.00005 | Predicted Protein |
| 49 | TRINITY_DN2764_c0_g1 | 0.03 | 0 | 0 | 27.4 | 33.19 | 14.81 | -8.65 | 0.00002 | WRKY transcription factor 6 (WRKY DNA-binding protein 6, AtWRKY6) |
| 50 | TRINITY_DN3685_c0_g1 | 0.54 | 0.06 | 0 | 173.37 | 95.47 | 98.32 | -8.63 | 0.00025 | Predicted Protein |

**Table 4S.** Top 50 upregulated genes from copper susceptible samples compared to water controls in *Pinus banksiana*

| Rank | Gene ID | Sus 1 | Sus 2 | Sus 3 | Water 1 | | Water 2 | Water 3 | logFC | Adj. P. Value | UniProt Description |
| --- | --- | --- | --- | --- | --- | --- | --- | --- | --- | --- | --- |
| 0 | TRINITY_DN2786_c0_g1 | 670.58 | 354.91 | 364.1 | 0 | 0 | | 0 | 13.15 | 8.45E-06 | Predicted Protein |
| 1 | TRINITY_DN1628_c0_g1 | 1238.41 | 1180.01 | 590.75 | 0 | | 0.32 | 0 | 12.80 | 2.48E-05 | Trypsin inhibitor [Cleaved into: Trypsin inhibitor chain A; Trypsin inhibitor chain B ] |
| 2 | TRINITY_DN258556_c0_g1 | 248.22 | 356.86 | 541.07 | 0 | | 0 | 0 | 12.77 | 1.88E-05 | Predicted Protein |
| 3 | TRINITY_DN1368_c0_g1 | 1712.84 | 2189.53 | 1629.49 | 0 | | 1.3 | 0.07 | 12.73 | 2.67E-06 | Predicted Protein |
| 4 | TRINITY_DN5716_c0_g1 | 2481.86 | 3248.79 | 5881.21 | 0 | | 7.03 | 0.41 | 12.53 | 6.37E-04 | Predicted Protein |
| 5 | TRINITY_DN2832_c0_g1 | 358.6 | 494.32 | 122.28 | 0 | | 0 | 0 | 12.49 | 3.11E-05 | Predicted Protein |
| 6 | TRINITY_DN5391_c1_g1 | 496.98 | 221.21 | 166.02 | 0 | | 0 | 0 | 12.43 | 3.94E-05 | Predicted Protein |
| 7 | TRINITY_DN57079_c0_g1 | 223.25 | 327.76 | 184.27 | 0 | | 0 | 0 | 12.22 | 1.57E-07 | Predicted Protein |
| 8 | TRINITY_DN5965_c1_g1 | 799.48 | 842.36 | 692.63 | 0.33 | | 0 | 0 | 12.12 | 2.95E-06 | Predicted Protein |
| 9 | TRINITY_DN50999_c1_g1 | 333.04 | 156.25 | 133.24 | 0 | | 0 | 0 | 11.95 | 1.73E-05 | Predicted Protein |
| 10 | TRINITY_DN55243_c0_g1 | 115.54 | 286.47 | 226.14 | 0 | | 0 | 0 | 11.88 | 1.05E-05 | Predicted Protein |
| 11 | TRINITY_DN1520_c0_g1 | 1020.58 | 623.65 | 1427.83 | 0.02 | | 0.65 | 0 | 11.86 | 1.18E-03 | Trypsin inhibitor [Cleaved into: Trypsin inhibitor chain A; Trypsin inhibitor chain B ] |
| 12 | TRINITY_DN5795_c0_g1 | 851.63 | 854.34 | 325.97 | 0 | | 0.84 | 0 | 11.83 | 2.09E-04 | Predicted Protein |
| 13 | TRINITY_DN7061_c1_g1 | 194.74 | 291.48 | 98.46 | 0 | | 0 | 0 | 11.82 | 2.94E-06 | Predicted Protein |
| 14 | TRINITY_DN8563_c1_g1 | 131.04 | 322.9 | 115.04 | 0 | | 0 | 0 | 11.71 | 4.77E-06 | Predicted Protein |
| 15 | TRINITY_DN4524_c0_g3 | 113.8 | 426.21 | 83.05 | 0 | | 0 | 0 | 11.63 | 9.67E-05 | Predicted Protein |
| 16 | TRINITY_DN257933_c1_g1 | 201.53 | 169.25 | 77.24 | 0 | | 0 | 0 | 11.48 | 4.34E-06 | Predicted Protein |
| 17 | TRINITY_DN3536_c0_g1 | 122.8 | 169.34 | 90.42 | 0 | | 0 | 0 | 11.28 | 3.13E-07 | Predicted Protein |
| 18 | TRINITY_DN237688_c0_g1 | 211.18 | 103.61 | 72.51 | 0 | | 0 | 0 | 11.25 | 1.84E-05 | Predicted Protein |
| 19 | TRINITY_DN7685_c0_g1 | 509.79 | 347.25 | 228.34 | 0 | | 0.41 | 0 | 11.24 | 6.78E-05 | Predicted Protein |
| 20 | TRINITY_DN14732_c0_g1 | 372.88 | 97.02 | 31.35 | 0 | | 0 | 0 | 11.18 | 1.92E-03 | Predicted Protein |
| 21 | TRINITY_DN12750_c0_g1 | 166.37 | 64.69 | 116.96 | 0 | | 0 | 0 | 11.11 | 3.61E-05 | Predicted Protein |
| 22 | TRINITY_DN2463_c0_g1 | 818.33 | 234.67 | 289.22 | 0 | | 0.04 | 0.11 | 11.02 | 2.24E-03 | Predicted Protein |
| 23 | TRINITY_DN3069_c0_g1 | 313.54 | 458.61 | 155.11 | 0 | | 0.37 | 0 | 10.98 | 6.68E-05 | Predicted Protein |
| 24 | TRINITY_DN2221_c0_g1 | 664.03 | 226.26 | 181.23 | 0 | | 0.68 | 0 | 10.90 | 1.96E-03 | Predicted Protein |
| 25 | TRINITY_DN3092_c0_g1 | 661.05 | 923.49 | 459.73 | 0.14 | | 0.35 | 0 | 10.88 | 6.47E-06 | Glucan endo-1,3-beta-glucosidase, acidic isoform, EC 3.2.1.39 ((1->3)-beta-glucan endohydrolase, (1->3)-beta-glucanase) (Beta-1,3-endoglucanase) |
| 26 | TRINITY_DN4477_c1_g1 | 81.97 | 139.91 | 53.7 | 0 | | 0 | 0 | 10.74 | 1.70E-06 | Predicted Protein |
| 27 | TRINITY_DN1315_c0_g1 | 763.2 | 1448.5 | 490.5 | 0.14 | | 1.09 | 0 | 10.71 | 7.58E-05 | Beta-glucosidase 12, EC 3.2.1.21 |
| 28 | TRINITY_DN1315_c0_g1 | 763.2 | 1448.5 | 490.5 | 0.14 | | 1.09 | 0 | 10.71 | 7.58E-05 | Furcatin hydrolase, FH, EC 3.2.1.161 |
| 29 | TRINITY_DN1315_c0_g1 | 763.2 | 1448.5 | 490.5 | 0.14 | | 1.09 | 0 | 10.71 | 7.58E-05 | Non-cyanogenic beta-glucosidase, EC 3.2.1.21 |
| 30 | TRINITY_DN1315_c0_g1 | 763.2 | 1448.5 | 490.5 | 0.14 | | 1.09 | 0 | 10.71 | 7.58E-05 | Beta-glucosidase 27, Os8bglu27, EC 3.2.1.21 |
| 31 | TRINITY_DN1315_c0_g1 | 763.2 | 1448.5 | 490.5 | 0.14 | | 1.09 | 0 | 10.71 | 7.58E-05 | Beta-glucosidase 11, Os4bglu11, EC 3.2.1.21 |
| 32 | TRINITY_DN1315_c0_g1 | 763.2 | 1448.5 | 490.5 | 0.14 | | 1.09 | 0 | 10.71 | 7.58E-05 | Beta-glucosidase 24, Os6bglu24, EC 3.2.1.21 |
| 33 | TRINITY_DN1315_c0_g1 | 763.2 | 1448.5 | 490.5 | 0.14 | | 1.09 | 0 | 10.71 | 7.58E-05 | Beta-glucosidase 13, Os4bglu13, EC 3.2.1.21 |
| 34 | TRINITY_DN3685_c0_g2 | 458.68 | 319.42 | 270.94 | 0.01 | | 0.58 | 0 | 10.68 | 4.23E-05 | Copia protein (Gag-int-pol protein) [Cleaved into: Copia VLP protein; Copia protease, EC 3.4.23.- ] |
| 35 | TRINITY_DN89721_c0_g1 | 78.43 | 118.71 | 57.37 | 0 | | 0 | 0 | 10.67 | 6.77E-07 | Predicted Protein |
| 36 | TRINITY_DN2391_c0_g1 | 858.87 | 316.78 | 250.41 | 0.79 | | 0 | 0 | 10.66 | 2.47E-03 | Predicted Protein |
| 37 | TRINITY_DN129489_c0_g1 | 108.85 | 67.2 | 66.28 | 0 | | 0 | 0 | 10.65 | 2.71E-06 | Predicted Protein |
| 38 | TRINITY_DN705_c0_g1 | 396.92 | 327.8 | 168.62 | 0 | | 0.2 | 0.08 | 10.61 | 5.98E-05 | Aldehyde oxidase GLOX, EC 1.2.3.1 (Glyoxal oxidase, VpGLOX) |
| 39 | TRINITY_DN6211_c0_g1 | 84.27 | 82.59 | 60.21 | 0 | | 0 | 0 | 10.56 | 4.71E-07 | Predicted Protein |
| 40 | TRINITY_DN77041_c1_g1 | 119.39 | 88.54 | 34.89 | 0 | | 0 | 0 | 10.55 | 1.71E-05 | Predicted Protein |
| 41 | TRINITY_DN2914_c0_g1 | 125.88 | 118.78 | 89.54 | 0 | | 0.03 | 0 | 10.53 | 1.15E-06 | Protein TIFY 10b, OsTIFY10b (Jasmonate ZIM domain-containing protein 7, OsJAZ7) (OsJAZ6) |
| 42 | TRINITY_DN2914_c0_g1 | 125.88 | 118.78 | 89.54 | 0 | | 0.03 | 0 | 10.53 | 1.15E-06 | Protein TIFY 3B (Jasmonate ZIM domain-containing protein 12) |
| 43 | TRINITY_DN34759_c0_g1 | 322.78 | 155.49 | 180.5 | 0 | | 0.56 | 0 | 10.40 | 2.41E-04 | Predicted Protein |
| 44 | TRINITY_DN6089_c0_g1 | 198.66 | 247.89 | 178.38 | 0 | | 0.52 | 0 | 10.38 | 1.19E-05 | Predicted Protein |
| 45 | TRINITY_DN4195_c0_g1 | 66.81 | 67.79 | 59.62 | 0 | | 0 | 0 | 10.34 | 5.60E-07 | Predicted Protein |
| 46 | TRINITY_DN1537_c0_g1 | 83.24 | 55.67 | 49.47 | 0 | | 0 | 0 | 10.29 | 2.21E-06 | Predicted Protein |
| 47 | TRINITY_DN9955_c0_g1 | 73.8 | 59.93 | 51.33 | 0 | | 0 | 0 | 10.27 | 9.89E-07 | Predicted Protein |
| 48 | TRINITY_DN40097_c0_g1 | 364.94 | 781.26 | 1375.23 | 0 | | 3.39 | 0.69 | 10.26 | 1.28E-03 | Predicted Protein |
| 49 | TRINITY_DN4694_c0_g2 | 103.71 | 56.82 | 31.28 | 0 | | 0 | 0 | 10.22 | 1.99E-05 | Predicted Protein |
| 50 | TRINITY_DN141140_c2_g1 | 355.97 | 220.27 | 133.34 | 0 | | 1.24 | 0 | 10.22 | 3.52E-04 | Predicted Protein |

**Table 5S.** Top 50 downregulated genes from copper susceptible samples compared to water controls in *Pinus banksiana*

| Rank | Gene ID | Sus 1 | Sus 2 | Sus 3 | Water 1 | Water 2 | Water 3 | logFC | Adj. P Value | UniProt Description |
| --- | --- | --- | --- | --- | --- | --- | --- | --- | --- | --- |
| 0 | TRINITY_DN293_c0_g1 | 0.02 | 0 | 4.27 | 83.3 | 131.42 | 164.86 | -11.03 | 5.76E-05 | Delta-selinene-like synthase, chloroplastic, PsTPS-Sell, EC 4.2.3.76 |
| 1 | TRINITY_DN293_c0_g1 | 0.02 | 0 | 4.27 | 83.3 | 131.42 | 164.86 | -11.03 | 5.76E-05 | Alpha-humulene synthase, EC 4.2.3.104 (Terpene synthase TPS-Hum, PgTPS-Hum) |
| 2 | TRINITY_DN293_c0_g1 | 0.02 | 0 | 4.27 | 83.3 | 131.42 | 164.86 | -11.03 | 5.76E-05 | Delta-selinene synthase, EC 4.2.3.71, EC 4.2.3.76 (Agfdsel1) |
| 3 | TRINITY_DN5038_c0_g2 | 0.21 | 0.18 | 1.49 | 147.03 | 102.4 | 230.91 | -10.45 | 0.000203 | Predicted Protein |
| 4 | TRINITY_DN1269_c0_g1 | 0 | 0 | 0.13 | 32.25 | 13.5 | 37.33 | -10.30 | 0.000142 | Predicted Protein |
| 5 | TRINITY_DN4890_c0_g1 | 0 | 0 | 0 | 15.59 | 12.05 | 25.46 | -10.25 | 7.16E-06 | Predicted Protein |
| 6 | TRINITY_DN2314_c0_g1 | 0.03 | 0.05 | 0.05 | 40.88 | 14.56 | 52.04 | -10.14 | 0.000719 | Predicted Protein |
| 7 | TRINITY_DN8038_c0_g1 | 0.16 | 0.3 | 0.98 | 122.65 | 81.13 | 105.38 | -10.02 | 7.75E-05 | Probable aquaporin PIP2-8 (Plasma membrane intrinsic protein 2-8, AtPIP2;8) (Plasma membrane intrinsic protein 3b, PIP3b) |
| 8 | TRINITY_DN26931_c0_g1 | 1.66 | 0 | 0 | 65.61 | 45.82 | 36.39 | -9.96 | 0.000315 | Probable aquaporin PIP2-8 (Plasma membrane intrinsic protein 2-8, AtPIP2;8) (Plasma membrane intrinsic protein 3b, PIP3b) |
| 9 | TRINITY_DN10618_c0_g1 | 0 | 0 | 0 | 17.92 | 13.75 | 10 | -9.96 | 9.77E-06 | Predicted Protein |
| 10 | TRINITY_DN4059_c0_g1 | 0.07 | 0 | 0 | 20.09 | 12.1 | 19.58 | -9.78 | 1.71E-05 | Predicted Protein |
| 11 | TRINITY_DN159567_c0_g1 | 0 | 0 | 0 | 11.89 | 6.31 | 15.47 | -9.60 | 2.54E-05 | WAT1-related protein At5g07050 |
| 12 | TRINITY_DN34182_c0_g1 | 0 | 0 | 0 | 11.56 | 9.9 | 10.5 | -9.59 | 2.74E-06 | Predicted Protein |
| 13 | TRINITY_DN129749_c0_g1 | 0 | 0 | 0 | 9.6 | 9.98 | 11.12 | -9.52 | 1.52E-06 | Predicted Protein |
| 14 | TRINITY_DN129793_c0_g1 | 0 | 0 | 0 | 8.28 | 13.37 | 9.36 | -9.48 | 1.16E-06 | Putative UPF0481 protein At3g02645 |
| 15 | TRINITY_DN2617_c0_g1 | 0 | 0 | 0 | 12.84 | 6.76 | 7.48 | -9.34 | 2.38E-05 | Predicted Protein |
| 16 | TRINITY_DN11362_c0_g1 | 0 | 0.76 | 0.54 | 39.53 | 30.88 | 57.71 | -9.33 | 0.000138 | Predicted Protein |
| 17 | TRINITY_DN4176_c0_g1 | 0.47 | 0 | 0.44 | 61.56 | 18.42 | 66.16 | -9.31 | 0.003861 | Chalcone synthase, EC 2.3.1.74 (Naringenin-chalcone synthase) |
| 18 | TRINITY_DN2507_c0_g1 | 0 | 1.1 | 0 | 32.43 | 13.12 | 23.67 | -9.23 | 0.000645 | Predicted Protein |
| 19 | TRINITY_DN113586_c0_g1 | 0 | 0 | 0 | 7.25 | 5.74 | 13.28 | -9.21 | 1.26E-05 | Predicted Protein |
| 20 | TRINITY_DN1400_c0_g1 | 0.07 | 0.06 | 0.21 | 24.93 | 11.67 | 26.83 | -9.20 | 0.000262 | Subtilisin-like protease SBT5.6, EC 3.4.21.- (Subtilase subfamily 5 member 6, AtSBT5.6) |
| 21 | TRINITY_DN26605_c0_g1 | 0 | 0 | 0 | 6.61 | 7.11 | 10.35 | -9.13 | 2.92E-06 | Predicted Protein |
| 22 | TRINITY_DN7878_c0_g1 | 0.4 | 1.2 | 1.38 | 124.44 | 78.86 | 175.53 | -9.11 | 5.18E-05 | Predicted Protein |
| 23 | TRINITY_DN1514_c0_g1 | 0.27 | 0.75 | 1.81 | 82.92 | 99.69 | 154.44 | -9.11 | 4.49E-06 | Germin-like protein 8-14 (Germin-like protein 1) (Germin-like protein 5, OsGER5) |
| 24 | TRINITY_DN21458_c0_g1 | 0 | 0 | 0 | 8.31 | 6.63 | 7.86 | -9.11 | 4.53E-06 | Predicted Protein |
| 25 | TRINITY_DN69830_c0_g4 | 0.03 | 0.09 | 0.04 | 10.13 | 7.29 | 18.59 | -9.10 | 4.00E-05 | Predicted Protein |
| 26 | TRINITY_DN3730_c0_g1 | 0.24 | 0.3 | 0.46 | 67.3 | 33.24 | 49.71 | -9.09 | 0.000334 | Cytochrome P450 720B2, EC 1.14.-.- (Cytochrome P450 CYPB) |
| 27 | TRINITY_DN14060_c0_g1 | 0 | 0 | 0 | 6.65 | 5.95 | 9.46 | -9.02 | 4.58E-06 | Predicted Protein |
| 28 | TRINITY_DN4395_c0_g1 | 0 | 0 | 0 | 5.51 | 5.99 | 11.79 | -9.02 | 7.91E-06 | Predicted Protein |
| 29 | TRINITY_DN1838_c0_g1 | 0.11 | 0 | 0.32 | 8.28 | 25.04 | 47.89 | -9.00 | 0.001175 | Predicted Protein |
| 30 | TRINITY_DN6372_c0_g1 | 0 | 0 | 0.08 | 6.68 | 8.88 | 21.69 | -8.99 | 5.86E-05 | Predicted Protein |
| 31 | TRINITY_DN4245_c0_g1 | 0 | 0 | 0.26 | 6.69 | 11.32 | 27.52 | -8.93 | 0.000179 | Predicted Protein |
| 32 | TRINITY_DN66388_c0_g1 | 0 | 0 | 0 | 7.93 | 3.28 | 10.77 | -8.93 | 7.89E-05 | Predicted Protein |
| 33 | TRINITY_DN15910_c0_g1 | 0.15 | 0.09 | 0.97 | 49.33 | 33.41 | 37.21 | -8.88 | 0.000109 | Predicted Protein |
| 34 | TRINITY_DN20386_c0_g2 | 0 | 0.49 | 0 | 27.65 | 4.28 | 16.45 | -8.85 | 0.004171 | Predicted Protein |
| 35 | TRINITY_DN5204_c0_g1 | 0.18 | 0 | 0.27 | 5.5 | 35.56 | 46.25 | -8.84 | 0.005204 | Predicted Protein |
| 36 | TRINITY_DN12418_c0_g1 | 0.08 | 0 | 2.51 | 36.43 | 22.5 | 33.82 | -8.83 | 0.000338 | Predicted Protein |
| 37 | TRINITY_DN20386_c0_g1 | 0 | 0 | 0 | 7.77 | 6 | 5.13 | -8.83 | 9.59E-06 | Predicted Protein |
| 38 | TRINITY_DN17857_c0_g1 | 0.2 | 0 | 0.72 | 32.67 | 13.34 | 53.26 | -8.82 | 0.002403 | Predicted Protein |
| 39 | TRINITY_DN51950_c1_g1 | 0 | 0 | 0 | 6.24 | 5.15 | 7.26 | -8.80 | 5.33E-06 | Predicted Protein |
| 40 | TRINITY_DN51306_c0_g1 | 0.19 | 0.97 | 7.2 | 159.73 | 61.66 | 89.23 | -8.80 | 0.0004 | Predicted Protein |
| 41 | TRINITY_DN175470_c0_g2 | 0 | 0 | 0 | 7.48 | 3.57 | 7.41 | -8.76 | 3.34E-05 | Predicted Protein |
| 42 | TRINITY_DN6927_c0_g2 | 0 | 0 | 0.06 | 9.83 | 5.96 | 11.75 | -8.75 | 3.78E-05 | Metacaspase-9, AtMC9, EC 3.4.22.- [Cleaved into: Metacaspase-9 subunit p20; Metacaspase-9 subunit p10 (Metacaspase 2f, AtMCP2f) ] |
| 43 | TRINITY_DN3304_c0_g1 | 0 | 0 | 0 | 9.07 | 9.08 | 2.36 | -8.74 | 0.00018 | Predicted Protein |
| 44 | TRINITY_DN229927_c0_g1 | 0 | 0 | 0 | 7.04 | 3.93 | 6.56 | -8.71 | 1.85E-05 | Predicted Protein |
| 45 | TRINITY_DN20766_c0_g1 | 0.02 | 0 | 0.1 | 10.17 | 5.57 | 10.32 | -8.69 | 4.75E-05 | Subtilisin-like protease SBT1.7, EC 3.4.21.- (Cucumisin-like serine protease) (Subtilase subfamily 1 member 7, AtSBT1.7) (Subtilisin-like serine protease 1, At-SLP1) |
| 46 | TRINITY_DN16281_c0_g1 | 0 | 0 | 0 | 3.97 | 4.71 | 10.6 | -8.69 | 1.70E-05 | Histone H3.2 (Histone H3.1) |
| 47 | TRINITY_DN100_c0_g2 | 0 | 0.41 | 0.08 | 17.78 | 10.18 | 22.45 | -8.69 | 0.000216 | Predicted Protein |
| 48 | TRINITY_DN647_c0_g2 | 0 | 0 | 0.32 | 15.17 | 6.47 | 13.72 | -8.67 | 0.000266 | Purple acid phosphatase 3, EC 3.1.3.2 |
| 49 | TRINITY_DN56109_c0_g1 | 0 | 0 | 0 | 4.74 | 3.74 | 10.12 | -8.67 | 2.53E-05 | Predicted Protein |
| 50 | TRINITY_DN3133_c0_g1 | 0 | 0 | 0.53 | 23.93 | 5.75 | 13.65 | -8.65 | 0.001948 | Predicted Protein |
