# Supplementary material for: Gene expression profiling of Jack Pine (Pinus banksiana) under copper stress: Identification of genes associated with copper resistance
Source: PLoS One. 2024 Mar 7;19(3):e0296027. doi: 10.1371/journal.pone.0296027 (PMC10919686; doi:10.1371/journal.pone.0296027)
Supplement: S2 File — (DOCX) [file pone.0296027.s002.docx]

a)

b)

**Figure 1S.** Percentage of the top 100-upregulated transcripts in *Pinus banksiana* a) resistant genotypes compared to susceptible genotypes and b) susceptible samples compared to water categorized by cellular compartment. They were grouped by Gene Ontology terms within the cellular compartment using Omicsbox/BLAST2GO. Terms with lower than 2% of total gene expression were combined together and assigned the label “other”.

a)

b)

**Figure 2S.** Percentage of the top 100-downregulated transcripts in *Pinus banksiana* from a) resistant genotypes compared to the susceptible genotypes and b) susceptible genotypes compared to water controls. They were grouped by Gene Ontology terms within the Cellular Component category using Omicsbox/BLAST2GO. Terms with lower than 2% of total gene expression were combined together and assigned the label “other”.
